# Rearing pigs with play opportunities: the effects on disease resilience in pigs experimentally inoculated with PRRSV

**DOI:** 10.3389/fvets.2024.1460993

**Published:** 2024-09-17

**Authors:** Karolína Steinerová, John C. S. Harding, Sarah E. Parker, Heather L. Wilson, Arthur Nery Finatto, Yolande M. Seddon

**Affiliations:** ^1^Department of Large Animal Clinical Sciences, Western College of Veterinary Medicine, University of Saskatchewan, Saskatoon, SK, Canada; ^2^Vaccine and Infectious Disease Organization, International Vaccine Centre (VIDO-InterVac), Saskatoon, SK, Canada

**Keywords:** pig, play behaviour, positive emotions, porcine reproductive and respiratory syndrome virus (PRRSV), disease resilience, infectious challenge, positive animal welfare, swine

## Abstract

Positive emotions can reduce disease susceptibility during infectious challenges in humans, and emerging evidence suggests similar effects in farm animals. Because play behaviour may support a positive emotional state in pigs, this study investigates whether rearing pigs with regular intermittent play opportunities enhances disease resilience when challenged with porcine reproductive and respiratory syndrome virus (PRRSV). Litters were assigned to either play (PLY; *n* = 5 L) or control (CON; *n* = 4 L) treatments at birth. In PLY, play was promoted with extra space and enrichment items for three hours daily from five days of age (doa). At weaning (25 ± 2 doa; mean ± SD), 28 pigs (14/treatment) were selected for a disease challenge, based on weight, sex, and sow. The pigs were transported to a disease containment facility and at 43 ± 2 doa (day 0 post-inoculation, DPI) inoculated with PRRSV. Skin lesions, blood, rectal temperature, clinical signs, body weight, and behaviour were collected pre- and post-inoculation. Play opportunities for PLY continued every other day until euthanasia of all pigs at 65 ± 2 doa (22 DPI). PLY pigs exhibited fewer skin lesions following transport and throughout the infection compared to CON. Although the viral load did not differ between treatments, PLY pigs had a lower probability of experiencing moderate and severe respiratory distress, with a shorter duration. PLY also performed better throughout the infection, showing higher ADG and greater feed efficiency. The immune response differed as well. PLY pigs had fewer monocytes on 8 DPI than CON, with levels returning to baseline by 21 DPI, whereas CON levels exceeded baseline. Regardless of day of infection, lymphocyte counts tended to be lower in PLY than in CON, and white blood cells and neutrophils were also lower, but only in slow-growing pigs. PLY pigs continued to play during the infection, demonstrating less sickness behaviour and emphasizing the rewarding properties of play. Results suggest that PLY pigs were less affected by PRRSV and developed increased resilience to PRRSV compared to CON. This study demonstrates that rearing pigs in an environment supporting positive experiences through provision of play opportunities can enhance resilience against common modern production challenges, underscoring the value of positive welfare in intensive pig farming.

## Introduction

Positive emotions have been associated with improved health in humans (1, 2), and emerging evidence suggests similar effects in farm animals (3, 4). Emotions and immunity work in synergy, and there are indications that a positive affective state has a beneficial effect on disease resilience (4, 5). Resilience, defined as the ability of the animal to minimize the impact of environmental, social and disease challenges and quickly return to pre-challenge status (6), is imperative to sustain efficient pig production. An environment promoting positive experiences and the satisfaction of pigs’ behavioural needs may improve pig resilience against common stressful challenges such as disease (7), transport (8), and injury (9). In pigs, positive emotions could be facilitated by offering opportunities to engage in a rewarding activity promoted by a stimulus-rich environment (10). During co-infection with porcine reproductive and respiratory syndrome virus (PRRSV) and *Actinobacillus pleuropneumoniae* (APP), pigs reared in a stimuli-rich environment with substrates including straw, moist peat, wood shavings, destructible objects, and greater space with opportunities for increased social interaction between non-litter mates, had a lower PRRSV RNA concentration 8 days post-inoculation compared to conventionally-reared control pigs, suggesting faster viral clearance (7). Only one pig out of 14 pigs reared in the enriched environment developed macroscopic lung lesions compared to eight pigs reared under control conditions (7). Moreover, the histological evaluation of the lesions was less severe in the enriched group (7). Pigs reared in an alternative housing system (multi-suckling, delayed weaning (9 weeks of age), extra space, physical enrichment from jute sacks and straw at farrowing, and various chewable and durable enrichment with deep bedding post-weaning) demonstrated quicker recoveries from several challenges (8). The enriched pigs showed a lower response and/or returned to baseline faster following lipopolysaccharide (LPS) (cortisol, non-esterified fatty acids) and 2-h transport challenges (cortisol, NEFA, glucose), and had lower levels of hair cortisol following the period of challenges (8). Further, grow-finish pigs that were tasked with mastering a cognitively rewarding activity, where they had to discern individual acoustic signals to receive a feed reward, showed faster wound healing (9). These studies (7–9) highlight the beneficial effects of a rearing environment that can promote positive experience on pig metrics relevant to production and welfare.

However, pigs in the majority of global pig production are reared in restricted and barren environments with partly- or fully-slatted flooring and the facilitation of positive experiences through the provision of rootable substrates and extra space is limited. Play is a promising candidate to become a tool to promote positive experiences for pigs as it is often associated with perceived joy and fun in non-human animals (11, 12). Research on rough-and-tumble play in rats revealed an overlap of the play circuit with the reward system in the brain (13). A recent study reported that play can be promoted and sustained in pigs beyond the period of its natural expression [2–6 weeks, (14)] in a commercial setting regardless of extra space, as long as pigs were provided with a rotation of novel enrichment (15), making it an attainable approach for promoting positive experiences on conventional farms. Steinerová et al. (15) also suggested that grow-finish pigs perceived play positively, as shown by a greater expression of behaviours previously linked to positive (anticipation, ears forward and relaxed) and fewer ear postures linked to negative experiences (ears backward), compared to pigs without play promotion.

Disease outbreaks pose a significant threat to the sustainability and profitability of global pig production. PRRSV is especially challenging to tackle since once introduced into a herd, it can become endemic (16). Typical PRRSV clinical signs are preweaning mortality, respiratory disease, fever, reduced feed intake and growth in newborn and growing pigs, and abortions in pregnant sows (16–18). PRRSV has a tropism for the cells of monocytic lineage with macrophages with a CD163 receptor as the primary replication site (19). The infection is characterised by persistent viremia and a disruption of the usual cascade of immune reactions (16). The suppression and delayed secretion of innate cytokines (20), neutralising antibodies and dysfunction of natural killer cells (16, 21), delays the onset of adaptive immunity, making infected pigs more susceptible to secondary bacterial infections (22). The virus promotes immunosuppressive response by increased secretion of pro-inflammatory cytokines IL-10 (23), and TGF-β (24), together with poor induction or complete suppression of anti-inflammatory IFN-γ (25). PRRSV also disrupts the secretion of thyroid hormones, negatively affecting growth (26). Currently, PRRSV is the most economically significant disease for large-scale intensive pig production systems, with the estimated annual loss in growing pigs being $361.8 million in the U.S. alone (27).

The proposed functions of play behaviour range from the enhancement of social cohesion (28, 29), the development of cognitive skills by training for the unexpected (12), to improving the performance of various adult activities (30). However, how play opportunities influence resilience during a disease challenge is unknown. The objective of the current experiment was to identify whether rearing pigs with play opportunities improved their disease resilience when challenged with PRRSV. This was examined by measuring the PRRSV-relevant immune, clinical and behavioural response and performance of pigs reared with play opportunities and control pigs reared conventionally. If play opportunities were beneficial for disease resilience, the pigs reared with play opportunities would demonstrate less severe clinical signs and return to pre-challenge health status faster than the control pigs.

## Materials and methods

All experimental procedures were reviewed and approved by the University of Saskatchewan Animal Care and Use Committee, AUP protocol #20200022, in accordance with the guidelines of the Canadian Council on Animal Care. This study was conducted at Prairie Swine Centre (PSC), Saskatoon, Canada, and in the Animal Care Unit (ACU) in the Western College of Veterinary Medicine at the University of Saskatchewan, Saskatoon, Canada, from February 2023 to April 2023. The PSC herd is regularly tested for PRRSV and was confirmed PRRSV negative by ELISA and PCR in two serum and lung samples, respectively on July 7, 2022.

### Animals and housing

Nine litters (*n* = 127 piglets) born to first or multi-parity sows (Camborough Plus) were enrolled in the experiment. All sows were housed in one farrowing room and farrowed within the same week. Experimental day 0 (D0) was the date the first litter was born. Cross-fostering piglets to and from litters was allowed only within 48 h of farrowing within treatment. Each litter was housed in a farrowing pen with tri-bar galvanised flooring (total dimensions of farrowing pen area: 1.8 m x 2.4 m, sow crate within: 0.6 m x 2.2 m) with a heat lamp and a rubber mat in a hooded creep area), sow feeder, and one nipple drinker for the sow above the feed through and a second nipple drinker just off the floor for the sow and piglets. At 3 days of age, piglets were tail-docked, ear notched for identification and males castrated. A dose of NSAID (Metacam®, 0.4 mg/kg; Boehringer Ingelheim, ON, Canada) was given via intramuscular injection to all pigs at the time of processing. On the same day, a standardised point-source enrichment, consisting of two strands of chain (0.4 m long/strand), was installed in each farrowing pen. The enrichment was attached to the back of the pen on a side wall with a carabiner, with the ends of the chains just touching the floor. Throughout the trial, the temperature in the farrowing room was set to 18°C with a heated creep area for piglets. Creep feed (Starter 2 RWA-Veggie, crumbles, Masterfeeds, SK, Canada) meeting the nutrient requirements of swine published by the National Research Council (31) was provided to piglets in a creep feeder *ad-libitum*, starting on D25. Health checks were done twice daily (AM and PM), and any medical treatments were recorded. Sick piglets were treated with a farm-specific treatment protocol and stayed in the farrowing pen.

### Experimental design

#### Before disease challenge

**Figure 1:**
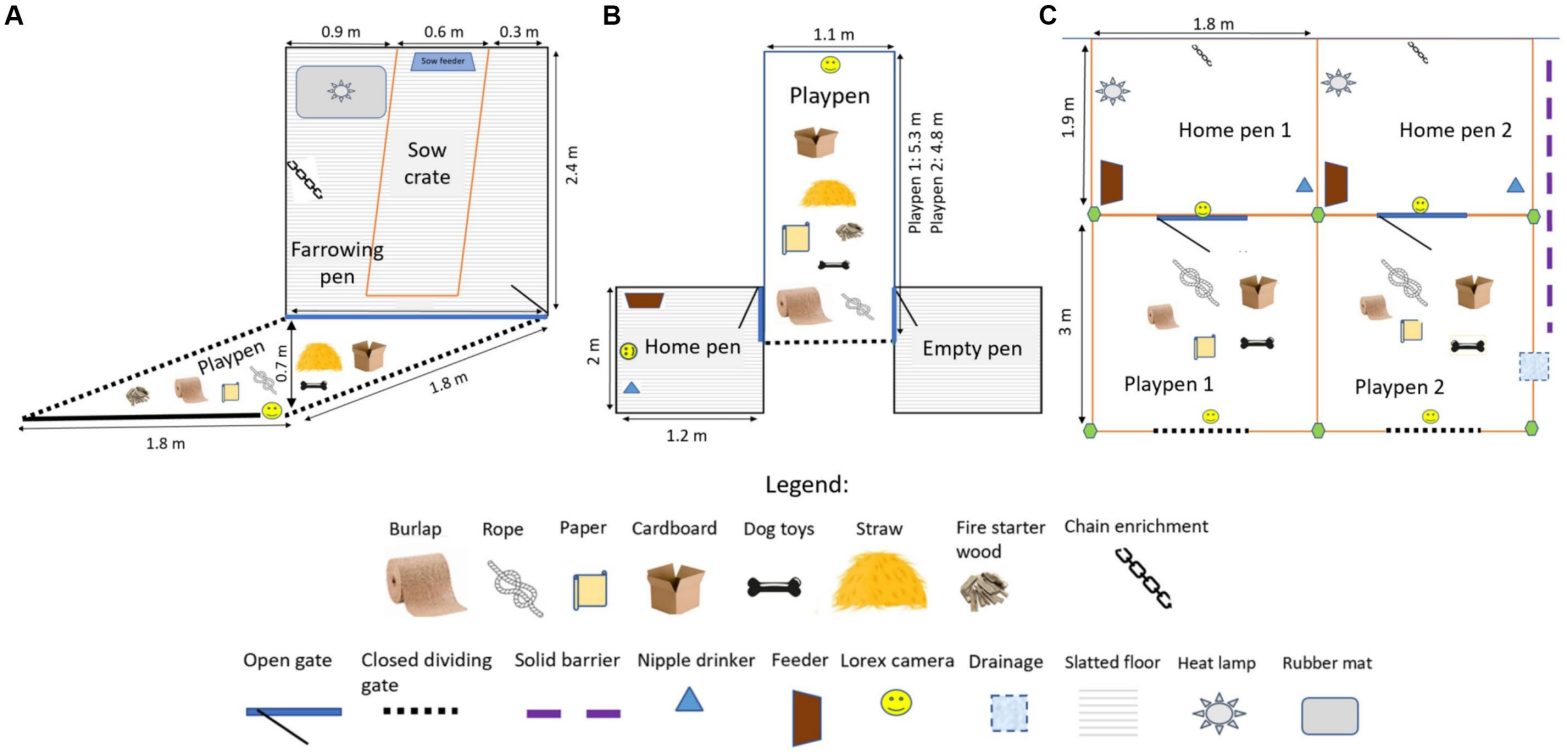
The layout of the home pens for Play (PLY) and Control (CON) treatments, and the playpens for PLY, is shown in **(A)** a farrowing room, **(B)** a nursery room, and **(C)** a disease containment facility at biosecurity level 2. Enrichment items were provided only in the playpens during the play sessions. The quantity of these items is not reflected here (for quantities, see Table 1).

The litters were assigned to either play (*n* = 5 L, 74 piglets, mean: 15 pigs/L, PLY) or control (*n* = 4 L, 53 piglets, mean: 13 pigs/L, CON) treatments at birth so that two litters in each treatment were born to multi-parity sows. The PLY treatment was reared with regular play opportunities from 5 days of age, whereas the CON treatment was reared under standard production conditions (farrowing pen: 0.3 m^2^/pig) without play opportunities. In PLY, play was promoted daily in a session from the AM to midday consisting of 3 h of continuous access to extra space in a playpen (1.8 m × 1.8 m; 0.5 m^2^/pig total space including the farrowing pen, Figure 1A) with the same seven types of destructible and durable enrichment items (Table 1). The playpens were situated behind the farrowing pen gate in the corridor and had solid flooring. A rubber mat was placed on the solid flooring for hygiene purposes and was cleaned daily. Before the onset of each play session, straw, cardboard, paper, toys, and wood were spread on the rubber mat in the playpen, and rope and burlap were fixed to a metal bar secured to the gate. Straw, paper and cardboard were replenished twice during the play session approximately after every hour, and rope with burlap was renewed daily. The entire litter was released to the playpen by opening the back gate, which also functioned as a barrier to prevent access to a neighbouring pen while maintaining access to the home farrowing pen. After 3 h, the piglets were guided back to the farrowing pen, the back gate was closed, and the enrichment was cleared from the pens. The farrowing pens of the PLY and CON treatments were situated on opposite sides of the room to avoid emotional contagion—the tendency to be behaviourally and physiologically affected by the emotional expression of others (32).

**Table 1:** Specifications of enrichment items (total amount, manufacturer) given during each play session to the Play treatment pigs in the farrowing room, nursery room, and disease containment facility at biosecurity level 2 (BSL2).

Enrichment item	Total amount in farrowing and nursery	Total amount in BSL2	Manufacturer
Straw	5 L	20 L	Simply Straw, fine cut 100% wheat straw, Lacombe, AB, CA
Cardboard	Thirty 0.1 m × 0.1 m pieces	Thirty 0.2 × 0.2 m pieces	Uline corrugated boxes, Pleasant Prairie, WI, USA
Construction paper	Thirty 0.1 m × 0.1 m pieces	Thirty 0.2 × 0.2 m pieces	Uline kraft paper sheets, Pleasant Prairie, WI, USA
Dog rubber toys (puppy size)	Three (1× bone, 1× connected circle, 1× ball)	Six (2× bone, 2× connected circle, 2× ball)	Dollarama Inc., Mount Royal, QC, CA
Fire starter wood	20 pieces	Not given	Vermont Castings Cherry Flavoured 100% Natural Smoking Wood Chips, Randolph, VT, USA
Cotton rope	Three strands of unravelled 0.8 m of ½ inch diameter	Six strands of unravelled 0.8 m of ½ inch diameter	Ropeshop.ca, Hamilton, ON, CA
Burlap	Three 0.1 m × 0.8 m pieces	Six 0.1 m × 0.8 m pieces	Uline burlap roll, Pleasant Prairie, WI, USA

Piglets were weighed at three and 23 ± 2 days of age (D26 of the experiment; mean ± S.D.).

#### Pen group formation for disease challenge

At weaning (25 ± 2 days of age, doa), new pen groups were formed, excluding fostered piglets, those with medical issues, and mortalities (four PLY piglets), resulting in 54 PLY and 46 CON piglets considered for the selection. In the PLY treatment, only piglets observed playing within the first 5 min of the play session at 21 doa were considered for pen groups.^1^ Thirty pigs were selected (15 pigs/treatment) for four pen groups within treatment, each of seven or eight pigs, balanced for similar D26 weight (PLY: 7.91 ± 0.80; CON: 7.88 ± 0.66; mean ± S.D., kg), sex, sow (4 sows/treatment), and additionally for PLY, the frequency of play at 21 doa (median: 14, range: 7–23). Twenty-eight pigs underwent a disease challenge (14 pigs/treatment) and two barrows served as negative control pigs (1 pig/treatment). The negative control pigs were included to ensure PRRSV-negative status of the pigs before inoculation and to verify that the pigs would have remained PRRSV-negative if not inoculated. The selected pigs were ear-tagged with prior administration of NSAID (Metacam®, 0.4 mg/kg). The pen groups were moved to a fully-slatted nursery pen (1.2 m × 2 m, 0.3 m^2^/pig) with one feed hopper (3 feeder spaces/hopper) and one nipple drinker. The playpens in the nursery room were accommodated in the central alleyway between pens (1.1 m × 4.8 m or 5.3 m; 0.8 m^2^/pig/playpen; Figure 1B). Pigs were vaccinated against porcine circovirus type 2 one day after weaning (1 mL/pig, CircoFlex, Boehringer Ingelheim Ltd., ON, Canada), fed *ad-libitum* with non-medicated starter feed (Starter 2 RWA-veggie pellets, VetaStart Pig Starter 4 short pellets, Masterfeeds, SK, Canada) until 9 weeks of age and then switched to grower feed (VetaGrow 16% Hog Grower Pellets, Masterfeeds, SK, Canada). The temperature in the nursery was 26°C during the first week, reduced weekly, and terminated at 20°C in week 10, following standard husbandry procedures.

#### Disease challenge

At 34 ± 2 days of age, the pigs were transported to a disease containment facility with biosecurity levels 1 and 2 (BSL1 and BSL2, Animal Care Unit, University of Saskatchewan). Upon arrival, a 7-day acclimation period started with daily play promotion for the PLY treatment. Twenty-eight pigs for the disease challenge were housed in groups of seven per pen (1.8 m × 1.9 m; 0.5 m^2^/pig/home pen) in the BSL2. The pigs remained in pen groups formed at weaning. The two negative control pigs were housed in one pen (2.2 m × 4.4 m; 4.8 m^2^/pig) in the BSL1. Each pen had solid flooring, a rubber mat (area), a heat lamp, one feed hopper (3 feeder spaces/hopper) and one nipple drinker. The home pens were cleaned daily (separate tool/treatment), and shavings were provided to soak up urine. The cleaning tools were rinsed with water and disinfected with 1% Virkon Solution (Virkon® S, Vetoquinol, Canada) between pens.

In the BSL2, the playpens (3 m × 1.8 m; 0.8 m^2^/pig/playpen) were adjacent to the home pens of the PLY treatment and separated by a gate connecting the playpen to the home pen that was left open during the play sessions (1.3 m^2^/pig total space for PLY during the play session). The amount of enrichment given per play session was increased (Table 1). A solid plastic barrier was installed between the two playpens to prevent visual and tactile contact and sharing of the enrichment between PLY pigs in each pen during the play sessions. The same barrier was erected between the home pens of PLY and CON to prevent visual contact (Figure 1C). After every play session, the playpens were cleaned and disinfected with 1% Virkon S. Similarly, as in the farrowing and nursery rooms, play was promoted daily in the AM to midday for 3 h, except for blood sampling days.

#### Inoculation with PRRSV

A PRRSV-2 isolate (NVSL 97–7895) was re-propagated on the MARC-145 cell line, thawed at room temperature, and diluted immediately before inoculation to 5 × 10^5^ TCID_50_ per mL in sterile phosphate-buffered saline (PBS) in a biosafety cabinet. The diluted inoculum was then immediately transported in a double container to the BSL2 while on ice. Following the acclimation period, at 43 ± 2 days of age (0-day post-inoculation, DPI), PLY and CON pigs in the BSL2 were inoculated with 1 × 10^6^ TCID_50_ PRRSV/pig, first intranasally to both nostrils (0.5 mL/nostril) with a nebulizer (MAD 110 Nasal Atomizer, Teleflex, IPN048827; Fisher FSTP9775029) and then intramuscularly (1 mL) in the neck. The negative control pigs in the BSL1 were sham-inoculated with PBS before the pigs in the BSL2.

#### Euthanasia and necropsy

All pigs were euthanized by anaesthetic overdose with Euthanyl (pentobarbital sodium, 390 mg/mL; Bimeda-MTC Animal Health Inc., ON, Canada) with prior sedation with Xylazine (100 mg/mL; Dechra Regulatory B.V., Netherlands) and Ketamine (100 mg/mL; Vètoquinol, QC, Canada) at 65 ± 2 days of age (22 DPI). Euthanasia was followed by necropsy.

### Animal measures

#### Behaviour and skin lesions

The duration of play (locomotor, social and object) and exploratory behaviour (Table 2) was scored with continuous sampling within the initial 10 min of the play sessions only in the PLY treatment at −2 (baseline), 3, 7, 11, 16, and 20 DPI. The scoring commenced after an experimenter exited the playpen and closed the gate. On the same days, to assess pig activity during the challenge, the frequency of active, inactive, and feeding behaviours (Table 2) in the PLY and CON treatments was assessed through instantaneous sampling within the first half of the play sessions at 5-min intervals (90 min, 18 scans/pig/DPI). Additionally, scans were collected at 10-min intervals when no play sessions were occurring for two 90-min periods without human presence in the pen, in the morning (between 7:30 and 9:30 AM) and evening (between 5:00 and 7:00 PM), totalling 180 min per day (18 scans/pig/DPI). In the chosen periods, human presence in the BSL2 was recorded only in the AM, and when it happened, an experimenter continued scanning but noted a person in the room. The pigs were individually marked with spray paint (Raidex GmbH, Dettingen/Erms, Germany) at least 2 h before video recording.

**Table 2:** Ethogram of play, exploratory, active, inactive, and feeding behaviours with the type of sampling (continuous, instantaneous) observed during the play sessions and in the AM and PM (only active, inactive, feeding behaviours) at −2, 3, 7, 11, 16, and 20 days post-inoculation (DPI).

Behaviour category (type of sampling)	*Behaviours* and postures	Description
Play behaviour (continuous^•^)	*Locomotor play*	Solitarily performing excitable, and energetic body movements, such as (i) jumping or whirling around to face in a different direction on the spot (pivot); (ii) running forward (gambolling); (iii) quickly laterally displacing the head and neck in both horizontal and vertical planes (head-toss); (iv) dropping rapidly from an upright position to a sitting or lying position (flop); (v) rolling its entire body from one lateral side to the opposite lateral side while lying on its back (rolling).
	*Social play*	Mutual pushing and head-knocking between two or more pigs using the snout, head, neck, and/or shoulders in an aroused/excited manner to engage in play of mild to moderate intensity.
	*Object play*	Engagement with objects by touching, chewing, shaking using the snout and/or mouth with/without carrying the objects in the mouth in an aroused/excited manner, and/or moving, kicking the objects with the limbs in an aroused/excited manner.
Exploratory behaviour (continuous^•^)	*Exploration*	Interacting with objects by nudging, pushing, sniffing, licking, and/or chewing using the snout and mouth.
Active behaviour (instantaneous^■^)	Standing	Supporting body weight on all four legs, weight on hooves and holding the torso and head upright without ambulation and contact with objects.
	Walking	Moving forward using all four legs for weight-bearing and ambulation, holding the torso and head upright without contact with objects and/or other pigs.
	Sitting	Hunches on the floor, hind legs are bent or stretched and do not bear weight, while the front legs are extended and support the body in an upright position.
	*Exploring*	Interacting with objects by nudging, pushing, rooting, sniffing, licking, and/or chewing using the snout and/or mouth while being in an upright position with/without ambulation
	*Playing*	Performing excitable, and energetic body movements, either solitary or mutually with conspecifics, with/without objects (locomotor, social and object play combined) while being in an upright position with ambulation.
	*Drinking*	The mouth is in contact with the drinker with/without ingestion of water while being in an upright position without ambulation.
	Active lying	Being in a prone position with the eyes open while interacting with objects by nudging, pushing, rooting, sniffing, licking, and/or chewing using the snout and/or mouth, with/without tactile contact with other pigs.
Inactive behaviour (instantaneous^■^)	*Inactive resting*	Being motionless in lateral recumbency with the eyes closed with/without tactile contact with other pigs.
Feeding behaviour (instantaneous^■^)	*Feeding*	The head is positioned in a feeding trough while the pig is standing, sitting, or in a prone position. Or the pig is chewing feed displaced from the feeder within a 1-m radius.

^•^Continuous sampling (scored only in the PLY treatment): within the first 10 min of the play sessions: 10 min/pig/DPI; analysed as duration. ^■^Instantaneous sampling (scored in the PLY and CON treatments): within the initial 90 min of the play sessions at 5-min intervals: 18 scans/pig/DPI; in the AM and PM at 10-min intervals for 180 min: 18 scans/pig/DPI; analysed as frequency.

All behaviours were videotaped with Lorex cameras (4K Ultra HD IP Security Camera, Lorex Technology, Markham, ON, Canada) in the farrowing room (one camera/two neighbouring home pens), the nursery room (one camera/home pen, one camera/two playpens) and the BSL2 room (one camera/pen; Figure 1). The behaviours were scored from the video recordings by one experimenter using the Observer software XT14 (version 14.2.1127, Noldus, Leesburg, VA, USA). The experimenter could not be blinded to the treatments due to clear distinctions between treatments in the experimental set up (playpens in PLY) and restricted number of trained personnel allowed to access to the disease containment facility.

Skin lesions were scored as a proxy measure of aggression (33) two days pre-weaning (age: D23 ± 2 (days); mean ± S.D.), one-day post-weaning (age: D26 ± 2), one day before transport (age: D33 ± 2), one day after transport (age: D35 ± 2), pre-inoculation (−2 DPI, age: D40 ± 2) and at the end of the trial (21 DPI, age: D64 ± 2). The body was divided into six regions: ears, face, front (the, shoulders, and front legs), middle (the body after the shoulders up to the frontal tip of the hind legs), rear (the hind legs), and tail^2^ [modified from (33)]. Each body region was scored individually and was assigned a score from 0 to 3: score 0 (none) = no lesions; score 1 (mild) = less than five superficial scratches; score 2 (moderate) = 5–10 superficial scratches and/or less than three deep wounds; score 3 (severe) = more than 10 superficial scratches and/or more than three deep wounds. A total body skin lesion score was calculated by summing all body region scores per pig and day (maximum score of 18/pig/day). One experimenter, who could not be blinded to the treatments, directly scored the skin lesions while standing outside the pen.

#### Rectal temperature and body weight

Rectal temperature (RT) was taken on 0 (baseline), 2, 4, 8, 13, 17, and 21 DPI using a digital thermometer with a flexible tip and a resolution of 0.1°C. On 0 DPI, a baseline RT was measured pre-inoculation. However, due to technical difficulties with the thermometer, the initial baseline data were discarded, and the baseline RT was recorded 3 h post-inoculation before the onset of detectable viremia [6–48 h post-exposure; (18)] using a new thermometer that was used thereafter. The pigs were weighted on 0, 8, 13, 17, and 21 DPI on a digital scale with a resolution of 0.1 kg. The ADG post-inoculation was calculated between each subsequent weigh period per pig.

#### Blood collection and clinical signs

Blood (serum, EDTA) was collected from the jugular vein with the pig restrained in a supine position on −1, 2, 4, 8, 13, 17, and 21 DPI. Tubes with EDTA were gently inverted 8–10 times to ensure thorough mixing with the anti-coagulant and stored on ice. Rectal temperature, body weight, and blood were collected between 8 and 10 AM in the aforementioned order.

To prevent cross-contamination, the negative control pigs in the BSL1 were blood sampled (serum, EDTA), and weighted and their RT was collected on −1 (blood) or 0 (weight, RT), 13, and 21 DPI before the pigs in the BSL2.

The pigs in the BSL2 were monitored for PRRSV clinical signs with scores assigned based on severity (0: not present, 4: severe) in the AM and PM. The negative control pigs in the BSL1 were monitored in the AM only by a separate team, from the first day in the acclimation period until 21 DPI. Monitored clinical signs included: respiratory distress (RD), coughing, responsiveness, appetite, colour of the skin, consistency of the faeces, body condition, and additionally lameness as a clinical sign not specific to PRRSV (see description in Supplementary Table 2).

#### Pen feed intake

Pen feed intake was recorded in the PM pre-inoculation on −8, −5, and −1 DPI, and post-inoculation on 2, 6, 9, 13, 16, 20, and 21 DPI. Feed intake was divided into periods: pre-inoculation (DPI −8 to −1; 8 days), one-week post-inoculation (DPI 0–6; 7 days), second-week post-inoculation (DPI 7–13; 7 days), third-week post-inoculation (DPI 14–21; 8 days), from which the average feed intake per pig per day in a given period was calculated. Feed-to-gain ratio (F:G) was calculated per pen (total (from 0 to 21 DPI) feed intake per pen/total gain per pen) and averaged per treatment.

#### Gross lung lesions

At necropsy, lungs were rinsed with water and carefully placed on a tray and their ventral and dorsal surface showing left and right cranial (CR), middle (M), caudal (CA), and accessory (A) lobes were photographed for later examination of pathomorphological changes. A consistent observer utilised a lung drawing from Halbur et al. (34) to shade areas on the lobes exhibiting the colour change observed in the photographs. Lung lesions typical of interstitial pneumonia and differing in severity with colour ranging from tan to dark red and purple (18) were identified. A 9 mm by 9 mm grid was placed on the shaded lung drawing to calculate the proportion of the affected lobes (number of shaded grid squares (with a precision of ¾ of a square)/total number of grid squares). This proportion was then multiplied with a pre-defined score assigned to each lobe [ventral left and right – CR: 10, M: 10, CA: 25, A: 5; dorsal left and right – CR: 10, M: 10, CA: 30; Halbur et al. (34)], resulting in an estimate of the percentage of the affected lobe, and thereafter summed to determine the total affected area of the lungs. Other characteristics of the gross lung lesions, such as the consistency of the lungs (slightly firm to rubbery) (18) were not possible to record from the photographs.

### Lab analyses

Immediately after the blood collection, whole blood (−1, 2, 4, 8, 13, 17, and 21 DPI) was submitted to Prairie Diagnostic Services (PDS) for a total count of white blood cells (WBC) and differential counts of lymphocytes, neutrophils, and monocytes counted in a haematology analyser (Advia 2120i, Siemens Healthcare Diagnostics, Erlangen, Germany). Serum was extracted from serum-separating tubes (Vacutainer® SST™) by centrifuging 1,500*g* for 10 min at 4°C, aliquoted to vials and stored at −80°C until further analysis. Serum samples (−1, 2, 4, 8, 13, 17, and 21 DPI) were analysed for total circulating triiodothyronine (T3), and PRRSV RNA. Triiodothyronine was quantified in PDS in the IMMULITE® 2000 Systems Analyser (Siemens Healthcare Diagnostics, Erlangen, Germany). The samples were assayed in duplicate with a calibration range of 0.61 to 9.2 nmol/L and analytical sensitivity of 0.29 nmol/L, with the protocol followed without any modifications.

#### Quantification of PRRSV RNA

The concentration of PRRSV strain NVSL 97–7895 RNA was determined using an in-house quantitative reverse transcription PCR assay (qRT-PCR). Each sample was individually assessed for the presence of target PRRSV RNA copies/mL. RNA was extracted from 140 μL of serum using the QIAamp Viral RNA mini kit (Qiagen Inc., Toronto, ON) according to the manufacturer’s instructions. The concentration (A260) and purity (A260/A280) of the extracted viral RNA were determined using spectrophotometry (NanoDrop 2000c, Thermo Fisher Scientific).

To quantify PRRSV RNA levels in the serum of pigs, a probe-based qRT-PCR assay previously described by Ladinig et al. (35) was employed. The primers and probe targeted the highly conserved region at the C-terminal end of ORF7 of NVSL 97–7895. The primer sequences were as follows: PRRS-2F primer 5′-TAA TGG GCT GGC ATT CCT-3′, PRRS-1R primer 5′-ACA CGG TCG CCC TAA TTG-3′, and the probe 5′-HEX-TGT GGT GAA TGG CAC TGA TTG RCA-BHQ2-3′. A dilution series (1.8 × 108 to 1.8 × 102 copies/μL) of HindIII linearized plasmid, pCR2.1TOPO-NVSL, containing a 446 bp sequence of ORF7, was used as a standard curve. The standards were run in triplicate on each PCR plate, while the tested samples were run in duplicate. All qRT-PCR reactions were performed on a 96-well plate (Hard-Shell 96-Well PCR Plate, Applied Biosystems), sealed with a Microseal ‘B’ PCR Plate Sealing Film (Applied Biosystems), and analysed on a Step-One Plus Real-Time PCR System (Bio-Rad Laboratories). Each qRT-PCR reaction consisted of 2 μL of sample or standard, 6 μL of RNAse-free water, 10 μL of iTaq Universal Probes 1-step kit (Bio-Rad Laboratories), 10 μM of PRRS-2F primer, 10 μM of PRRS-1R primer, 10 μM of PRRS-P1 probe, and 0.5 μL of iScript reverse transcriptase (Bio-Rad Laboratories). The thermocycling protocol included a reverse transcription step at 50°C for 30 min, followed by an initial activation step at 95°C for 10 min, and 40 cycles of denaturation (30 s at 95°C) and annealing/extension (30 s at 59°C). Individual samples were re-run if the cycle quantification (Cq) standard deviation between duplicates was >1.0 or if one of the duplicates had no cycle threshold (Ct) value. The results were reported as PRRSV RNA concentration per mL of serum. The limits of quantification were determined based on the least and most concentrated standards. Samples were considered negative if the target RNA was not detected or DNQ (detected, but not quantifiable).

### Statistical analyses

#### Standard statistical approach

Statistical analysis was performed on data from the experimentally inoculated pigs (*n* = 28) using the software STATA 17 (StataCorp LLC, TX, USA), while data from the negative control pigs (*n* = 2) were summarised descriptively. To test an underlying assumption for parametric testing, the normality of all data was assessed with the Shapiro–Wilk test. Based on the normality results and distribution evaluated in histograms, the data were analysed in regression with either linear, logistic, Poisson or negative binomial distributions with the pig as the experimental unit, except for feed intake analysed on a pen-level. Non-parametric Fisher’s exact test for independence was used to compare proportions where some categories had a few (≤10%) or no observations. Models were built in a forward stepwise inclusion of independent variables; important confounders were retained (20% change to effect for other variables). Interaction effects were assessed for inclusion. The Wald test estimated the overall significance of fixed effects. Interaction effects were also assessed visually, and three-way interactions were included only if they remained significant with relevant two-way interactions in the model. The fixed effects of treatment, sex, time variables (DPI, day, DPI period), birth weight, 0 DPI weight, ADG pre-weaning, and ADG pre-inoculation and their interaction effects were explored. Where collinearity existed between two variables, only one of the covariates could be included in the same model. If more than one collinear covariate was significant during model building, preference was given to the one with a stronger relationship defined by the coefficient of the dependent variable. The variables and their interaction effects were included in a final model if *p* ≤ 0.05 (the Wald test). Variability from clustering variables contributing to the dependent variables was assessed in an empty model with hierarchically nested pen and sow. Where repeated observations had been taken, the pig was included in the random statement to account for similarities within the pig. In linear and logistic mixed models, the proportion of total variance explained by clustered random effects was assessed with an intraclass correlation coefficient (ICC). Model fit was evaluated with the Akaike’s and Bayesian information criterion (AIC, BIC). Residuals of the final linear models were examined for normality and homoscedasticity. Model fit of logistic and Poisson regression was assessed with deviance residuals.

### Statistical analyses of animal measures

Factors affecting animal measures are summarised in Table 3.

**Table 3:** Factors affecting measured disease outcomes, behaviour, and performance in the Play treatment pigs reared with play opportunities and the Control treatment pigs experimentally inoculated with porcine reproductive and respiratory syndrome virus (PRRSV): overview of the fixed effects, interactions, and random effects included in the final multilevel multivariable regression models.

Outcome measures for separate models		Regression type	Fixed effects							Final interaction effects	Random effects		Pig^┼^
			Treatment	Time variable	Sex	Birth weight	0 DPI weight	ADG pre-weaning	ADG pre-inoculation		Pen	Sow	
Proxy of aggression	Total skin lesion score	Linear	●	Day	●					Treatment^X^Day			●
Systemic immune response	VL (AUC)	Linear	●								NA	NA	–
	WBC	Linear	●	DPI					●	Treatment^X^ADG pre-inoculation		●	●
	Lymphocytes	Linear	●	DPI									●
	Monocytes	Linear	●	DPI					●	Treatment^X^DPI			●
	Neutrophils	Linear	●	DPI					●	Treatment^X^ADG pre-inoculation			●
Clinical signs	RT	Linear	●	DPI				●				●	●
	Mild, moderate, severe RD (score ≥ 1)	Logistic	●	DPI		●				Treatment^X^Birth weight		●	●
	Moderate, severe RD (score ≥ 1.5)	Logistic	●	DPI				●			●	●	●
	Duration (days) of mild, moderate, severe RD (score ≥ 1)	Poisson	●			◌ confounder					NA	NA	NA
	Duration (days) of moderate, severe RD (score ≥ 1.5)	Poisson	●									●	–
Active behaviour	During play sessions	Negative binomial	●	DPI						Treatment^X^DPI			●
	AM	Poisson	●	DPI					●	Treatment^X^DPI			●
	PM	Poisson	●	DPI									●
Feeding behaviour (bouts)	During play sessions	Negative binomial	●										●
	AM & PM	Poisson	●	DPI				●			NA	NA	NA
Play and exploratory behaviours	Locomotor play	Negative binomial	NA	DPI									●
	Social play	Negative binomial	NA	DPI				●					●
	Object play	Negative binomial	NA	DPI									●
	Exploration	Negative binomial	NA	DPI									●
Performance	ADG pre-weaning^■^	Linear	●	NA								●	-
	ADG post-inoculation	Linear	●	DPI period						Treatment^X^DPI period			●
	T3	Linear	●	DPI	●	◌ confounder				Treatment^X^DPI			●
	Feed intake	Linear	●	DPI period							●		–
Post-mortem examination	Lung lesions	Linear	●								NA	NA	–

●Signifies inclusion in the model. ◌Denotes a confounder. ^┼^ Pig: where repeated observations had been taken, pig was included in the random statement to account for similarities within pig (“–” in a cell indicates that no repeated measures on a pig level were considered in the model). *RD: DPIs with ≤10% of the observations (<3 pigs observed) were excluded and analysed using Fisher’s exact test. ^■^Pre-weaning: additional confounder = sow parity. ADG, average daily gain; AUC, area under the curve; DPI, days post-inoculation; NA, random effects not applicable (fixed effects in the model only); RD, respiratory distress; RT, rectal temperature; T3, triiodothyronine; VL, viral load; WBC, white blood cells.

To explore the effects of play opportunities on performance before the PRRSV inoculation, ADG pre-weaning was analysed. Sow parity was added to a model, grouped as a two-level variable with sows having ≤1 (2 sows/CON, 3 sows/PLY) or ≥2 parities (2 sows/treatment).

The effect of treatment on aggressive behaviour was explored through total skin lesion score (the scores of all body regions, including tail,^1^ summed together per pig and day; max = 18) taken before and after the inoculation, and analysed a continuous variable in a mixed linear regression.

During the infection, the systemic immune responses, and clinical signs (Supplementary Table 2) were investigated through the analyses of viral load (VL), the number of white blood cells (WBC), lymphocytes, monocytes and neutrophils, triiodothyronine, gross lung lesions, medical treatments, rectal temperature (RT), respiratory distress (RD; incidence and duration in days), and responsiveness. Only clinical signs observed in the AM were considered for the analysis. Medical treatments were analysed using Fisher’s exact test. Medical treatments to treat fever were accounted for in an RT model and were not significant. Respiratory distress score was divided into four levels: none (score 0), mild (score 1), moderate (score 1.5, 2) and severe (score 2.5, 3). The duration of RD was calculated in two separate ways by adding the number of days each pig experienced: (1) any severity of RD (score ≥ 1), and (2) moderate and severe RD (score ≥ 1.5). Responsiveness scores were grouped into two categories, score 0 and score ≥1. Severe RD (score ≥ 2.5), its duration, and responsiveness were analysed using Fisher’s exact test due to infrequent occurrence. Other clinical signs were summarised descriptively due to infrequent occurrence.

The area under the curve (AUC) with a trapezoidal curve was calculated for PRRSV RNA concentrations over time (viral load) in serum. Then, the viral load (AUC) was analysed in a linear regression.

For the WBC count and its differentials, the average WBC reference intervals for swine obtained from PDS (Supplementary Table 3) were used to score individual pigs as either ‘0’ (within reference interval) or ‘1’ (outside of reference interval). To investigate whether the proportion of pigs with cell counts outside of the reference interval differed by treatment, data were explored in 2 × 2 contingency tables using Fisher’s exact test separately for each DPI.

Possible differences in active, feeding behaviours (scored with instantaneous sampling, Table 2) were explored between PLY and CON during the play sessions and in the AM/PM. Active behaviour in the AM was analysed only when pigs were not disturbed by a human in the room. The number of scans per pig and DPI was used as an exposure. To investigate the impact of PRRSV infection on the duration of locomotor, social and object play, and exploratory behaviour during the play sessions in PLY (scored via continuous sampling was explored). Total time observed per pig and DPI was used as an exposure.

Whether performance metrics were affected by the play opportunities during PRRSV infection, ADG post-inoculation and average feed intake were analysed in regression models. The feed-to-gain ratio was summarised descriptively.

One PLY pig died on 8 DPI due to a secondary bacterial infection, and its data were utilised until 8 DPI and then considered missing in all analyses, except for the ADG post-inoculation where all datapoints of the pig were excluded.^3^

**Figure 2:**
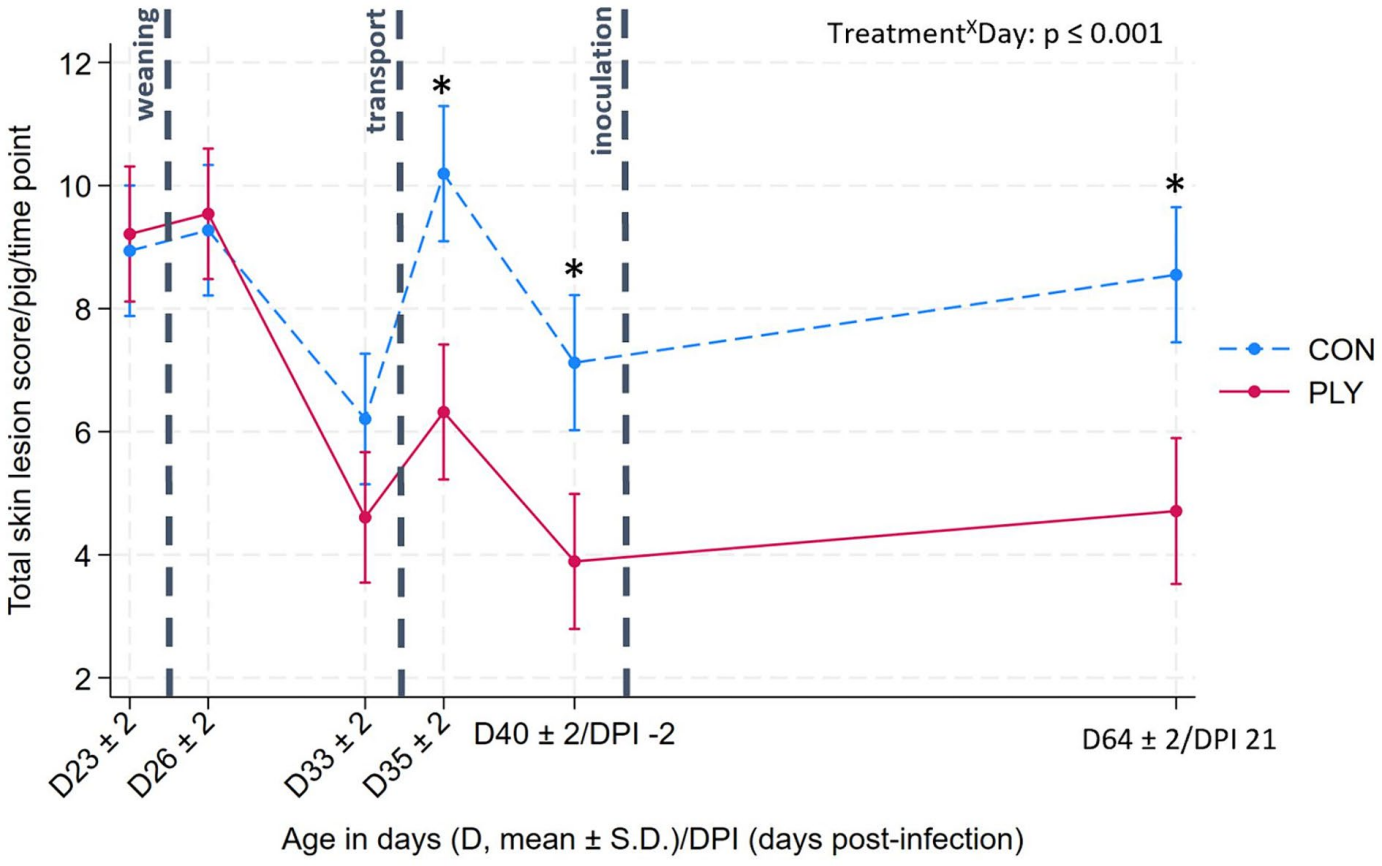
Total skin lesion scores in Play (PLY, solid line) and Control (CON, dashed line) treatments per pig (*n* = 28) at different time points: pre-weaning (age: D23 ± 2; mean ± S.D.), post-weaning (D26 ± 2), before transport (D33 ± 2), after transport (D35 ± 2), pre-inoculation (−2 DPI, D40 ± 2), and 21 DPI (D64 ± 2). Data are presented as predicted means with 95% confidence intervals. ‘X’ between two variables signifies an interaction effect. Significant differences between treatments within a day are denoted on the graph with an asterisk (*). The pairwise comparisons listed below have *p*-values less than or equal to the significant threshold (ST), adjusted using the Bonferroni correction to control the analysis-wise error. The type and number of comparisons are in italics and parentheses, respectively. Treatment^X^Day—*within treatment across consecutive days, pre-weaning vs. DPI21.* ST (18): *p* = 0.003. PLY: D26 ± 2 vs. 35, D33 ± 2 vs. 40, D64 ± 2 vs. 23. CON: D33 ± 2 vs. 26, D35 ± 2 vs. 33, D40 ± 2 vs. 35.

Results are presented as predicted means or counts of fixed or interaction effects and unadjusted lower and upper 95% confidence intervals (CIs). *p*-values are presented for the fixed effects. The results in the figures 2, 4, 5, 6, and 7 are reported over time (DPI, day, DPI period). Where significant interactions between specific variables were detected, comparisons are presented partitioned for variables in the interaction terms. To control overall *p*-values for multiple comparisons in specific models, significance thresholds (ST) were calculated using a Bonferroni correction (0.05/number of comparisons). Due to different interaction effects in specific models, different significance thresholds (ST) were calculated and are reported in the text and a footnote of a figure as ‘ST (number of comparisons): the threshold’s *p*-value’.

#### Interaction effect: division into weight categories

For the following interaction effects (denoted with ‘^X^’ between two variables), birth weight or weight gain was treated as a categorical variable and the pigs were categorised into: (1) slow- (*n* = 4 PLY, 5 CON pigs, mean = 0.28, range = 0.26–0.28; kg), medium- (*n* = 7 PLY, 3 CON pigs, 0.30, 0.29–0.31) and fast-growing (*n* = 3 PLY, 6 CON pigs, 0.33, 0.32–0.34) pigs pre-inoculation: Treatment^X^ADG pre-inoculation (WBC, neutrophils); (2) light (*n* = 7 PLY, 2 CON pigs, mean = 1.74, range = 1.65–1.80; kg), medium (*n* = 5 PLY, 6 CON pigs, 1.90, 1.85–2.00) and heavy (*n* = 2 PLY, 6 CON pigs, 2.16, 2.10–2.25) piglets at birth: Treatment^X^Birth weight (probability of any severity of RD).

## Results

The main and interaction effects relevant to treatment differences are summarised in Table 4. *p*-values of the main and interaction effects are also shown in the relevant figures. Pairwise comparisons of time points (across consecutive time points; baseline versus last time point) are reported in the footnotes of the figures. Other significant main effects (sex, time variable, ADG) are reported in Section 1 in Supplementary material.

**Table 4:** Overview of the effect of play opportunities on outcomes relating to disease, behaviour, and production measures in pigs inoculated with porcine reproductive and respiratory syndrome virus (PRRSV) as provided by separate multilevel multivariable regression models. The model output presented includes the *p*-value of treatment and interaction effects where applicable, along with pairwise comparisons and their threshold of significance, model fit (Akaike information criterion; AIC) and the intraclass correlation coefficient for random effects (ICC).

Outcome measures		Treatment	Interaction effect with treatment	*p*-value for threshold of significance for pairwise comparisons (number of comp.)	Type of pairwise comparisons	AIC	ICC
		*p*-value	*p*-value (overall, and for *pairwise comparisons*)				
Proxy of aggression	Total skin lesion score	0.727	Day overall: **≤0.001**	0.003 (18)	Between treatments within day & comparisons across consecutive days; bas vs. last^	767.652	Pig: 0.042
			*D35, D40 (-2DPI), D63 ± 2 (21DPI): all ≤ 0.001*				
Systemic immune response	VL (AUC)	0.362*	–	–	–	1,639.932	–
	WBC	**0.001**	ADG pre-inoculation overall: **0.002**	0.017 (3)	Between treatments within weight category	932.432	Sow: 0.140, pig: 0.446
			*Slow-growing pigs pre-inoculation: 0.001*				
	Lymphocytes	0.068	–	–	–	824.013	Pig: 0.354
	Monocytes	0.201	DPI overall: **0.006**	0.002 (21)	Between treatments within DPI & comparisons across consecutive DPIs; bas vs. last	115.658	Pig: 0.157
			*8DPI: 0.005^+^, 21DPI: ≤0.001*				
	Neutrophils	**0.005**	ADG pre-inoculation overall: **0.007**	0.017 (3)	Between treatments within weight category	811.634	Pig: 0.360
			*Slow-growing pigs pre-inoculation: 0.002*				
Clinical signs	RT	0.223	–	–	–	121.867	Sow: 0.136, pig: 0.182
	Mild, moderate, severe RD (score ≥ 1)	0.075	Birth weight overall: 0.086	0.017 (3)	Between treatments within weight category	238.972	Sow: 0.147, pig: 0.538
			*Light pigs at birth: 0.061*				
	Moderate, severe RD (score ≥ 1.5)	**≤0.001**	–	–	–	248.032	Pen: 1.19e−34, sow: 0.118, pig: 0.512
	Duration (days) of mild, moderate, severe RD (score ≥ 1)	0.098	–	–	–	153.153	NA
	Duration (days) of moderate, severe RD (score ≥ 1.5)	**≤0.001**	–	–	–	155.385	NA
Active behaviour	During play sessions	**≤0.001**	DPI overall: **≤0.001**	0.003 (18)	Between treatments within DPI & comparisons across consecutive DPIs; bas vs. last	827.354	NA
			*2, 3, 7, 11, 16, and 20DPI: all ≤ 0.001*				
	AM	0.683	DPI overall: **0.042**	0.003 (18)	Between treatments within DPI & comparisons across consecutive DPIs; bas vs. last	495.965	NA
			*11DPI: 0.003, 16DPI: ≤0.001*				
	PM	**≤0.001**	–	–	–	497.294	NA
Feeding behaviour (bouts)	During play sessions	0.870	–	–	–	544.937	NA
	AM & PM	0.092	–	–	–	566.251	NA
Performance	ADG pre-weaning	0.270	–	–	–	−348.553	Sow: 0.139
	ADG post-inoculation	0.940	DPI period overall: 0.063	0.004 (12)	Between treatments within DPI period & comparisons across consecutive DPI periods; bas vs. last	−166.739	Pig: 0.365
			*DPI period 3: 0.003, DPI period 4: 0.027^+^*				
	T3	**≤0.001**	DPI overall: **≤0.001**	0.002 (22)	Between treatments within DPI & comparisons across consecutive DPIs; bas vs. last	59.820	Pig: 0.166
			*−1DPI: ≤0.001*				
	Feed intake	0.139	–	–	–	14.193	Pen: 0.301
Post-mortem examination	Lung lesions	0.821	–	–	–	246.370	NA

Significant treatment and interaction effects are in bold (*p* ≤ 0.05). Locomotor, social, and object play, and exploratory behaviours were observed only in the PLY treatment, so comparison between the treatments was not possible (Model fit of those final models is in Supplementary material Section 3d). +Not significant in pairwise comparisons after Bonferroni correction but close to the threshold of significance. ^Definition of the abbreviation ‘bas vs. last’: comparison of baseline versus last time point. Where ‘–’ appears in a cell, no interaction effect was present. *Viral load: this model included all pigs (*n* = 28); for the predicted means and *p*-value from the model without the outlier from the CON treatment, see Supplementary material Section 3a. ADG, average daily gain; AUC, area under the curve; D, day; DPI, days post-inoculation; NA, ICC not applicable for this type of regression; RD, respiratory distress; RT, rectal temperature; T3, triiodothyronine; VL, viral load; WBC, white blood cells.

All pigs were PRRSV-negative pre-inoculation. The negative control pigs stayed PRRSV-negative post-inoculation and continued to maintain weight gain, and their count of WBC, lymphocytes, monocytes, and neutrophils stayed within the reference intervals (Supplementary Table 5).

### ADG pre-weaning

Pre-weaning ADG was not influenced by treatment (PLY: 0.23 [0.20, 0.25], CON: 0.24 [0.22, 0.27], predicted mean [95% CIs], kg; *p* = 0.270).

### Skin lesions

Total skin lesion scores were not different between the treatments pre-weaning (D23 ± 2; mean age in days ± S.D.), post-weaning (D26 ± 2) and before transport (D33 ± 2), but in the latter, the scores were lower than on previous days (Figure 2). After transport (D35 ± 2), the score increased in both treatment groups but was lower in PLY, remaining consistent with pre-transport levels, compared to CON. Pre-inoculation (−2 DPI), the score declined again with PLY maintaining a lower score than CON until the end of the experiment. At 21 DPI, PLY, but not CON, had a lower skin lesion score compared to pre-weaning (Figure 2).

### Viral load

**Figure 3:**
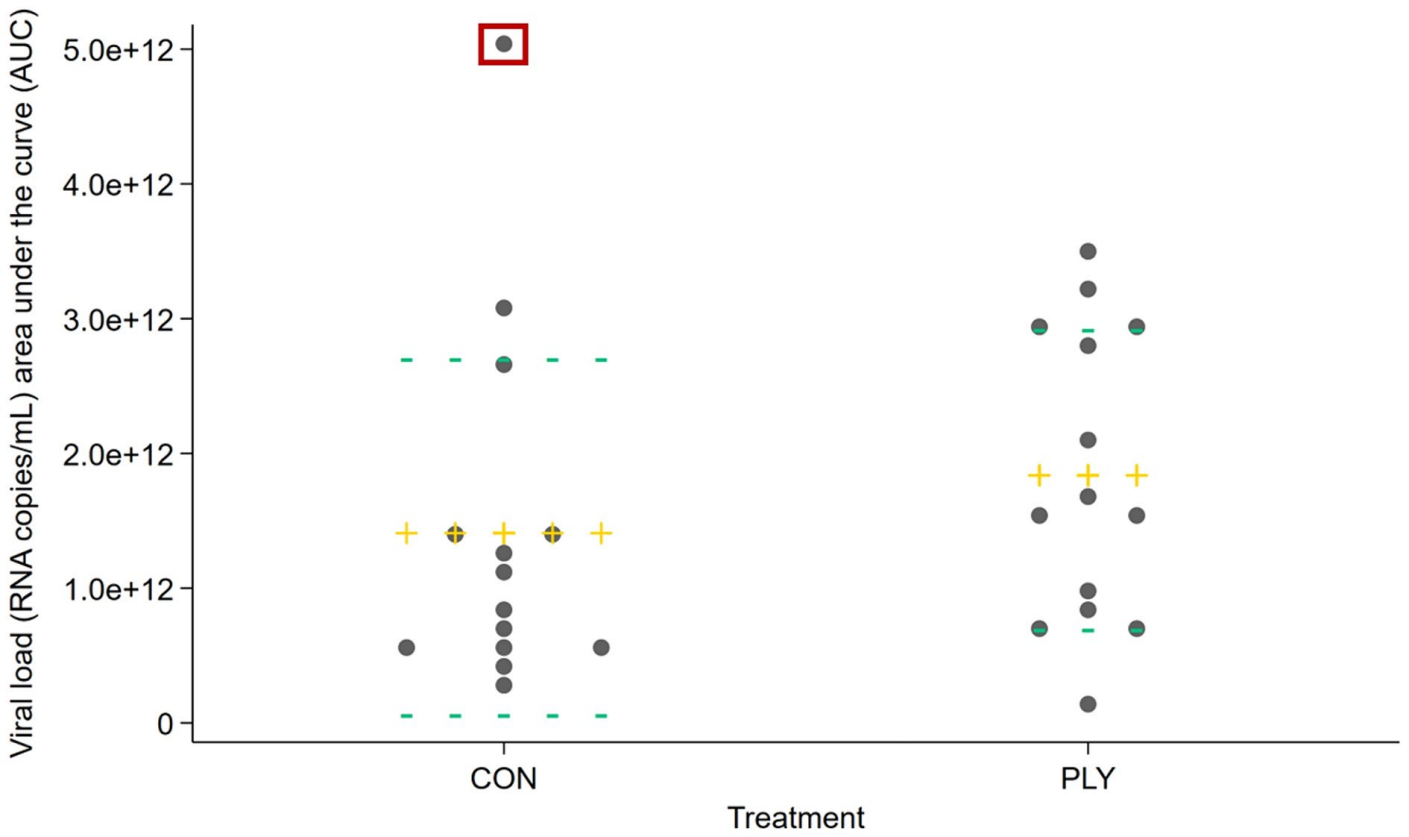
Viral load (RNA copies/mL) as area under the curve (AUC) of RNA copies between −1 and 21 days post-inoculation in Play (PLY) and Control (CON) treatments (*n* = 28). Data are presented as observed. Each grey dot represents a pig. The outlier from the CON treatment group is indicated by a red box. The mean is depicted as a yellow cross (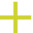), and the 25th and 75th percentiles are shown as green horizontal lines (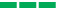).

Viral load was compared over time (area under the curve, AUC) among treatments. A descriptive summary of the data is shown in Figure 3. With all pigs considered (*n* = 28), there was no significant difference between PLY and CON (*p* = 0.362). After the removal of one outlier from the CON treatment (Figure 3, datapoint in a red box), PLY treatment pigs tended to have a higher AUC viral load (*p* = 0.077). The predicted means with and without the outlier are shown in Supplementary material Section 3a.

### Systemic immune response

**Figure 4:**
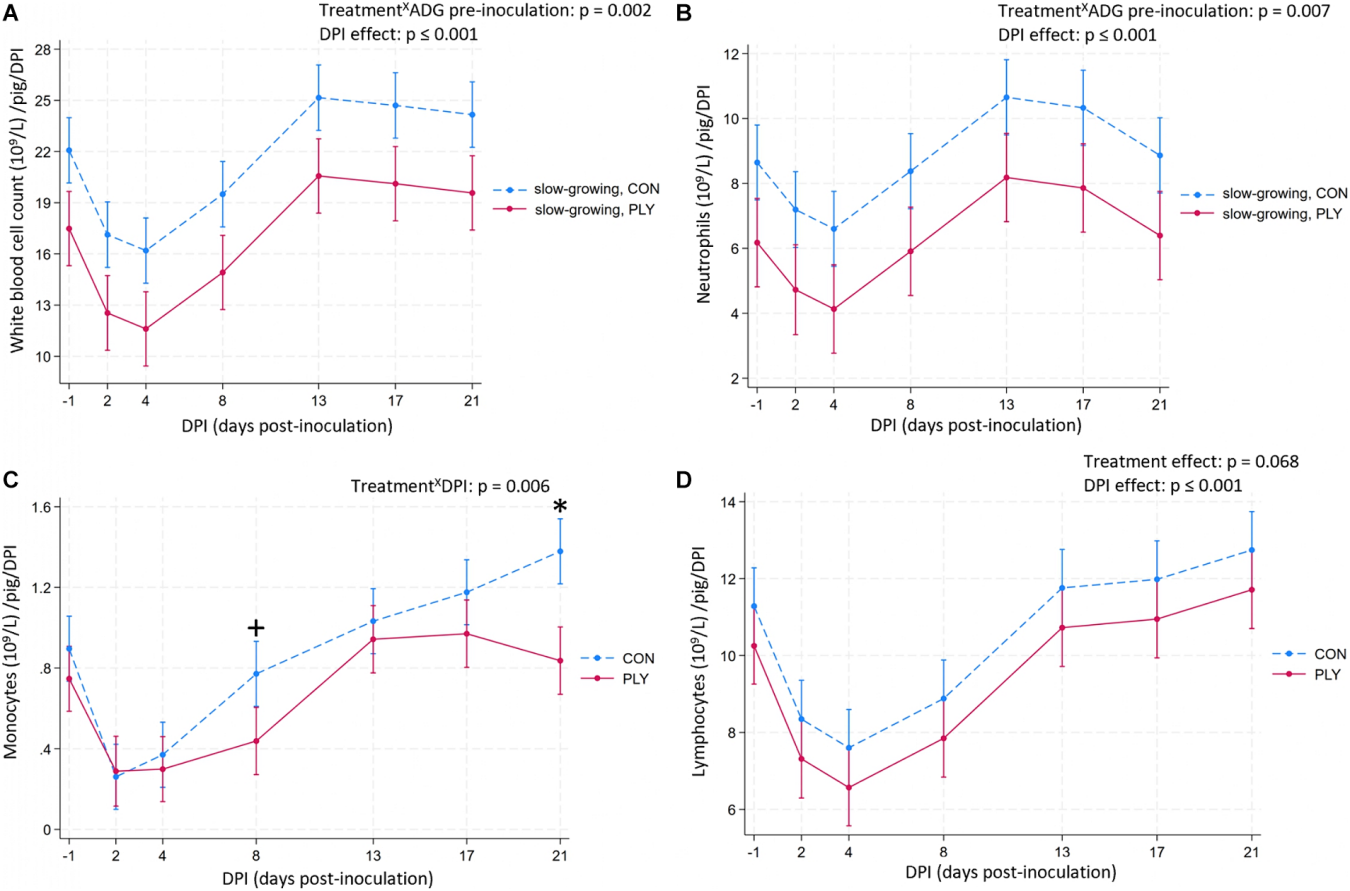
Total count of white blood cells (WBC) and neutrophils in slow-growing pigs pre-inoculation (WBC and neutrophils, 10^9^/L; **A,B**, respectively), monocytes (10^9^/L; **C**), lymphocytes (10^9^/L; **D**) in Play (PLY, solid line) and Control (CON, dashed line) treatments per pig (*n* = 28) on −1, 2, 4, 8, 13, 17, and 21 days post-inoculation (DPI). Data are presented as predicted means and 95% confidence intervals. ‘X’ between two variables signifies an interaction effect. Significant differences within DPI are denoted on the graph c with an asterisk (*). Weight categories based on ADG pre-inoculation shown on the graphs **(A,B)**: slow- (*n* = 4 PLY, 5 CON pigs, mean = 0.28, range = 0.26–0.28; kg), medium- (*n* = 7 PLY, 3 CON pigs, 0.30, 0.29–0.31) and fast-growing (*n* = 3 PLY, 6 CON pigs, 0.33, 0.32–0.34) pigs pre-inoculation: Treatment^X^ADG pre-inoculation (WBC, neutrophils). All weight categories are shown in Supplementary Figure 2. Graphs **(A,B,D)**: No interaction effect of treatment with time variable is present, thus, the presented values are summarised over all values (both treatments show the same DPI to DPI change). The pairwise comparisons listed below have *p*-values less than or equal to the significant threshold (ST), adjusted using the Bonferroni correction to control the analysis-wise error. The type and number of comparisons are in italics and parentheses, respectively. **(A)** DPI effect—*across consecutive days, baseline DPI-1 vs. 21*. ST (7): *p* = 0.007. DPI-1 vs. 2, DPI4 vs. 8, DPI8 vs. 13, DPI-1 vs. 21. **(B)** DPI effect—*across consecutive days, baseline DPI-1 vs. 21.* ST (7): *p* = 0.007. DPI-1 vs. 2, DPI4 vs. 8, DPI8 vs. 13, DPI17 vs. 21. **(C)** Treatment^X^DPI—*within treatment across consecutive DPIs, baseline DPI-1 vs. 21.* ST (21): *p* = 0.002. CON: DPI-1 vs. 2, DPI2 vs. 4, DPI-1 vs. 21, PLY: DPI-1 vs. 2, DPI8 vs. 13. +*p* = 0.005. **(D)** DPI effect—*across consecutive days, baseline DPI-1 vs. 21.* ST (7): *p* = 0.007. DPI-1 vs. 2, DPI4 vs. 8^+^ (*p* = 0.008), DPI8 vs. 13, DPI-1 vs. 21. ^+^close to the ST.

Slow-growing PLY pigs pre-inoculation had lower WBC and neutrophil counts compared to slow-growing CON pigs (Figures 4A,B), while these cell counts for medium- and fast-growing pigs did not differ (Supplementary Figure 2). The WBC count decreased until 4 DPI, then bounced back on 13 DPI to exceed the baseline levels, remaining constant until 21 DPI (Figure 4A). The quantity of neutrophils decreased following the inoculation, reaching peak levels at 13 DPI, thereafter declining, and returning to the baseline by 21 DPI (Figure 4B).

The numbers of monocytes decreased from the baseline at 2 and 4 DPI in both treatments and started to rebound at 8 DPI, but were numerically lower in PLY compared to CON. At 21 DPI, while PLY pigs returned to the baseline, CON exceeded the baseline and remained higher than PLY (Figure 4C).

The number of lymphocytes tended to be lower in PLY pigs. Lymphocyte quantities decreased at 2 DPI, increased at 4 DPI, and stabilised from 13 DPI to 21 DPI, returning to the pre-inoculation levels (Figure 4D).

No difference was seen in the proportion of pigs within and outside values of the reference interval (Supplementary Table 2) in the WBC count and its differentials (Supplementary Table 3).

### Rectal temperature and clinical signs

**Figure 5:**
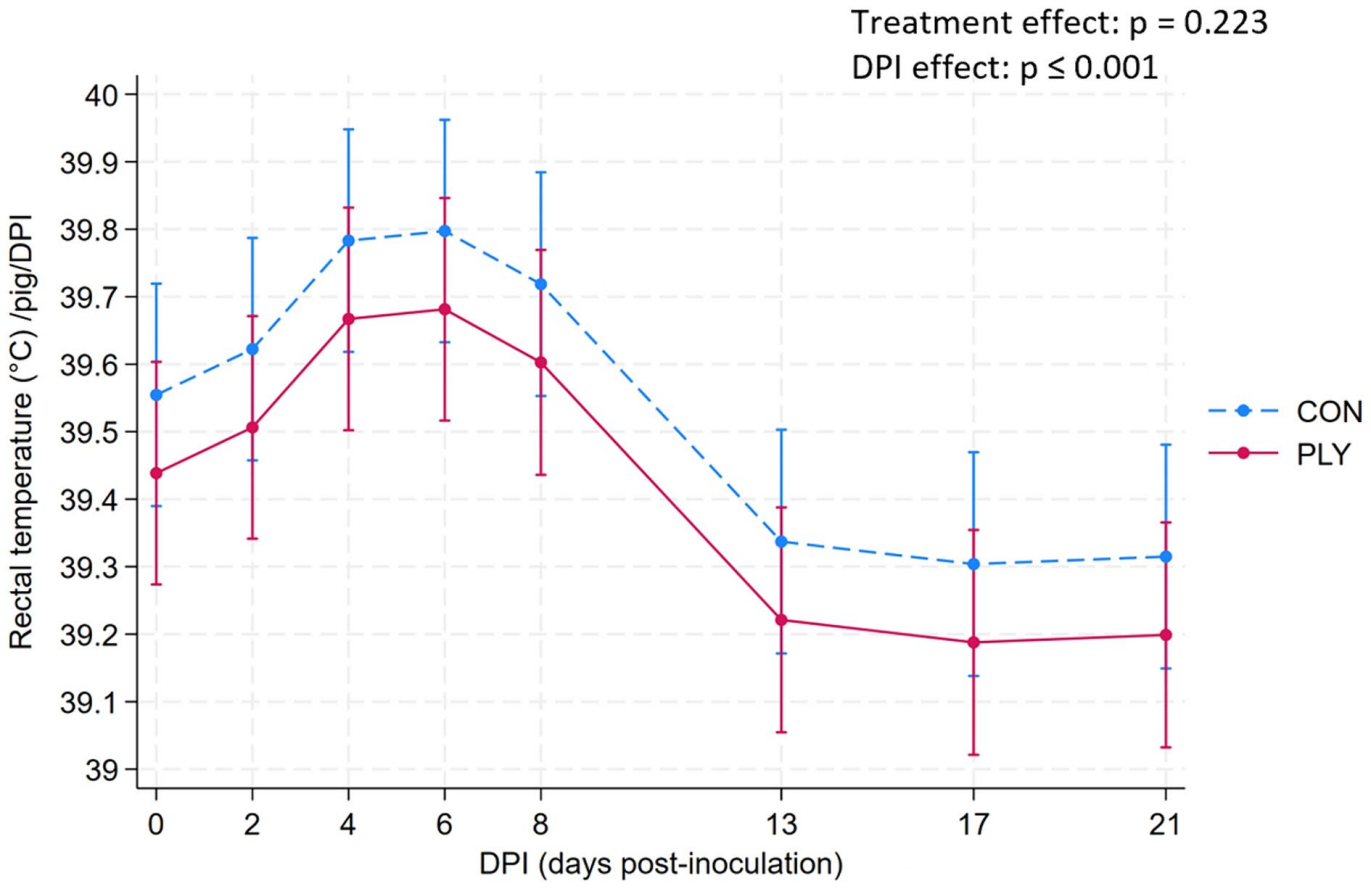
Rectal temperature (°C) in Play (PLY, solid line) and Control (CON, dashed line) treatments per pig (*n* = 28) on 0, 2, 4, 8, 13, 17, and 21 days post-inoculation (DPI). Data are presented as predicted means and 95% confidence intervals. ‘X’ between two variables signifies an interaction effect. No interaction effect of treatment with time variable is present, thus, the presented values are summarised over all values (both treatments show the same DPI to DPI change). The pairwise comparisons listed below have p-values less than or equal to the significant threshold (ST), adjusted using the Bonferroni correction to control the analysis-wise error. The type and number of comparisons are in italics and parentheses, respectively. DPI effect—*across consecutive days, baseline DPI0 vs. 21.* ST (8): *p* = 0.006. DPI8 vs. 13, DPI0 vs. 21.

Rectal temperature (RT) did not differ between PLY and CON. Over consecutive DPIs, the RT increased only numerically until 6 DPI, decreased between 8 and 13 DPI, and remained lower at 21 DPI compared to the baseline levels (Figure 5). Details about the individualized treatments for clinical signs of fever are shown in Supplementary material Section 3b.

Mild and moderate respiratory distress (RD) was analysed from 8 and 10 DPIs, respectively, because of a few (≤10%) or no observations on previous days. Each pig experienced RD of any severity (mild, moderate, severe: score ≥ 1) for at least 1 day, whereas moderate and severe RD (score ≥ 1.5) was experienced by 11 PLY and 14 CON pigs for at least 1 day. Two PLY and nine CON pigs experienced severe RD (score ≥ 2.5) for at least 1 day. Severe RD started to manifest at 7 DPI in one PLY pig, followed by one pig from both treatments from 11 until 13 DPI. Thereafter, none of PLY pigs experienced severe RD, compared to two to four CON pigs between 14 and 19 DPI, and eight CON pigs on 20 and 21 DPI (*p* = 0.002 for 20 and 21 DPIs, Fisher’s exact).

Mild, moderate, and severe RD was experienced for numerically fewer days in PLY than in CON (PLY: 9.85 [8.02, 11.68], CON: 12.33 [10.34, 14.31], predicted mean [95% CIs], days; *p* = 0.098). Similarly, light CON piglets at birth had a numerically higher probability of experiencing mild, moderate, and severe RD than light PLY (CON light: 0.62 [0.48, 0.77], PLY light: 0.82 [0.68, 0.96]; *p* = 0.061 [ST (3): *p* = 0.017]), with no differences between medium and heavy pigs at birth. PLY pigs suffered from moderate and severe RD significantly fewer days compared to CON (PLY: 4.12 [2.76, 5.48], CON: 11.58 [8.51, 14.64], predicted mean [95% CIs], days; *p* ≤ 0.001). PLY pigs had also a significantly lower probability of suffering from moderate and severe RD compared to CON (PLY: 0.35 [0.18, 0.52], CON: 0.91 [0.83, 0.99], predicted probability [95% CIs]; *p* ≤ 0.001). Other clinical signs (Supplementary Table 1) were observed sporadically, and their descriptive summary is in Section 2 in Supplementary material.

### Activity, play and exploratory behaviour

**Figure 6:**
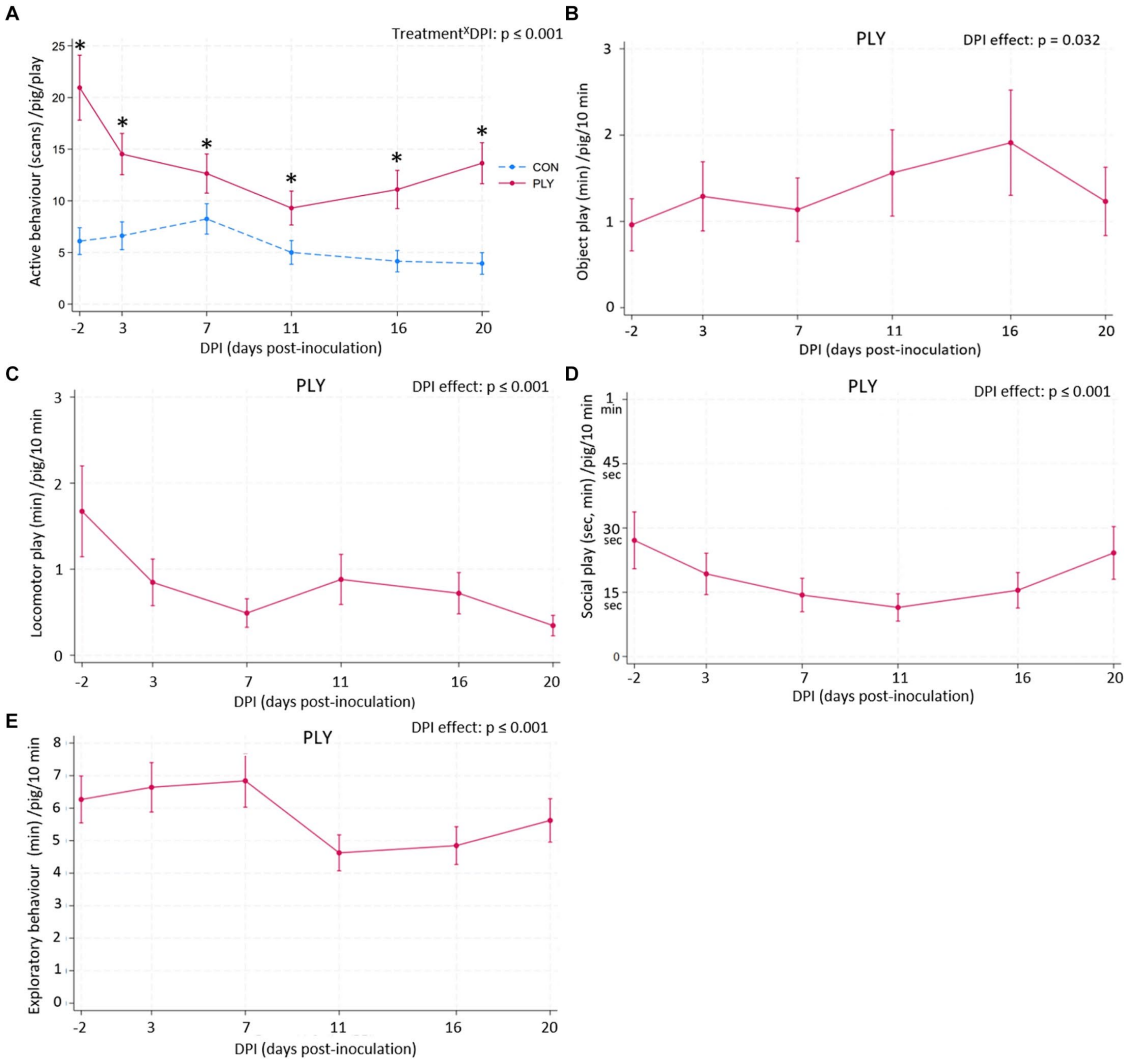
Active behaviour during the play sessions **(A)** in Play (PLY, solid line) and Control (CON, dashed line) treatments per pig (*n* = 28) and duration of object (min; **B**), locomotor (min; **C**) and social (sec, min; **D**) play and exploratory behaviour (min; **E**) in PLY (*n* = 14) within the initial 10 min of the play sessions on −2, 3, 7, 11, 16, and 20 days post-inoculation (DPI). Data are presented as predicted counts and 95% confidence intervals. ‘X’ between two variables signifies an interaction effect. Significant differences between the treatments within DPI are denoted on the graph **(A)** with an asterisk (*). The pairwise comparisons listed below have *p*-values less than or equal to the significant threshold (ST), adjusted using the Bonferroni correction to control the analysis-wise error. The type and number of comparisons are in italics and parentheses, respectively. Treatment^X^DPI—*within treatment across consecutive DPIs, baseline DPI-2 vs.* 20. **(A)** ST (18): *p* = 0.003. **(A)** PLY: DPI-2 vs. 3, DPI-2 vs. 20. CON: DPI11 vs. 17. DPI effect—*across consecutive DPIs, baseline DPI-2 vs.* 20. **(B–E)** ST (6): *p* = 0.008. **(C)** DPI-2 vs. 3, DPI16 vs. 20, DPI-2 vs. 20. **(E)** DPI7 vs. 11.

During the play opportunities, PLY pigs were more active pre-inoculation as well as during the infection compared to CON (Figure 6A). PLY pigs were most active pre-inoculation (on −2 DPI), then the activity declined until reaching the lowest level at 11 DPI and remained below the baseline at 21 DPI. CON pigs showed low activity levels both before and during the infection with lowest activity on 11 DPI (Figure 6A). Feeding behaviour during the play promotion (*p* = 0.870) and in the AM and PM (*p* = 0.092) was not affected by treatment. Results from active behaviour in the AM and PM are reported in Supplementary material Section 3c.

Within the initial 10 min of the play sessions, PLY pigs engaged in object, locomotor and social play to some extent in all days pre- and post-inoculation (Figures 6B–D). There was a time effect in object (Figure 6B) and social play (Figure 6D), however, no significant differences in pairwise comparisons were observed. PLY pigs engaged in locomotor play for the greatest duration of time pre-inoculation on −2 DPI with decreasing levels until 7 DPI and remaining constant in the second post-inoculation week. The lowest duration of time of locomotor play was performed at 20 DPI (Figure 6B). Exploratory behaviour was sustained in the first week post-inoculation, decreasing at 11 DPI, but 20 DPI was not different from the baseline (Figure 6E). Other details about the model outputs are in Supplementary material Section 3d.

### Performance and triiodothyronine

A datapoint of one CON pig from DPI period 4 was identified as an outlier with a biologically unlikely ADG (datapoint > 3 S.D. from the mean of the standard residuals; Supplementary Figure 1), and thus was excluded from this analysis.

**Figure 7:**
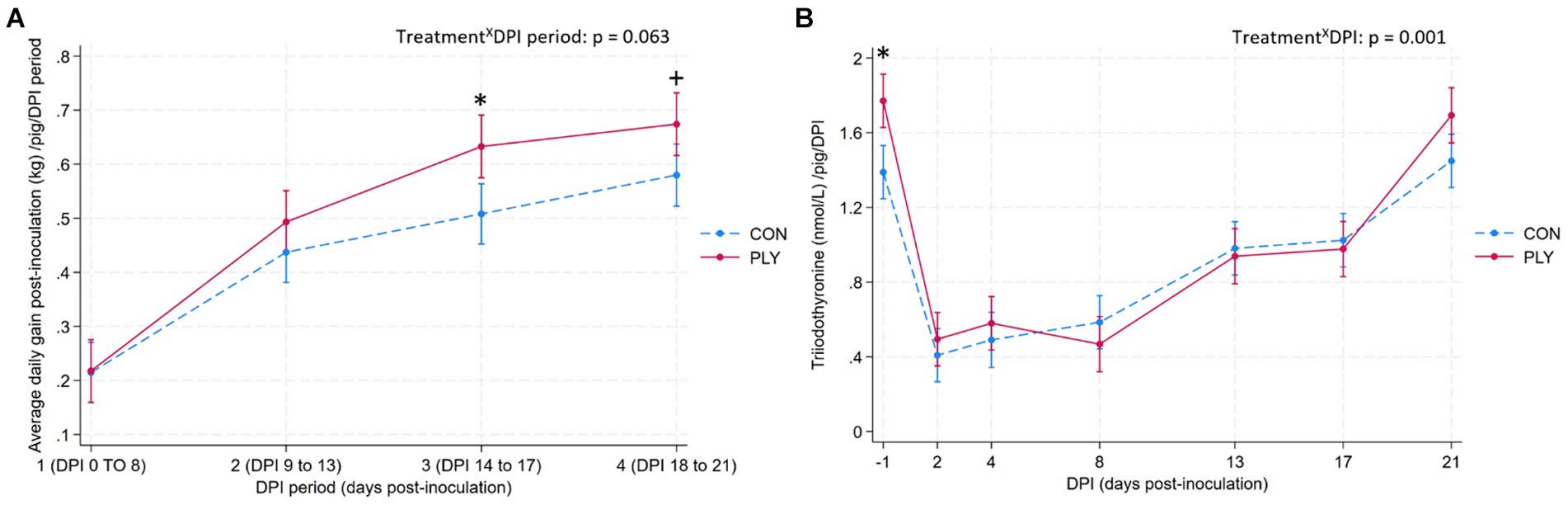
Average daily gain post-inoculation (kg, ADG; **A**) and triiodothyronine (nmol/L, T3; **B**) per pig in Play (PLY, solid line) and Control (CON, dashed line) treatments during periods 1 (DPI 0–8; days post-inoculation), 2 (DPI 8–13), 3 (DPI 13–17; *n* = 27^■^ in periods 1, 2, 3), and period 4 (DPI 17–21; *n* = 26^•^) for ADG, and on −1, 2, 4, 8, 13, 17, and 21 DPI for T3. Data are presented as predicted means and 95% confidence intervals. ‘X’ between two variables signifies an interaction effect. Significant differences between the treatments within DPI period or DPI are denoted on the graphs with an asterisk (*). The pairwise comparisons listed below have *p*-values less than or equal to the significant threshold (ST), adjusted using the Bonferroni correction to control the analysis-wise error. The type and number of comparisons are in italics and parentheses, respectively. **(A)** Treatment^X^DPI—*within treatment across consecutive DPI periods; baseline DPI period 1 vs. 4.* ST (12): *p* = 0.004. PLY: DPI period 1 vs. 2, 2 vs. 3, 1 vs. 4. CON: DPI period 1 vs. 2, 1 vs. 4. +*p* = 0.027. ^■^Datapoints of the deceased PLY pig from all DPI periods, and ^•^a datapoint of the CON outlier pig from DPI period 4 were excluded from this analysis. **(B)** Treatment^X^DPI—*within treatment across consecutive DPIs, baseline DPI-1 vs. 21*. ST (22): *p* = 0.002. PLY and CON: DPI-1 vs. DPI2, DPI8 vs. 13, DPI17 vs. 21.

PLY pigs started to have a higher ADG than CON from period 1, with a significant difference in period 3 between 13 to 16 DPI, and a greater numerical difference in period 4 between 17 and 20 DPI (Figure 7A). Both treatments increased their ADG between periods 1 and 2, but PLY pigs continued to show a higher difference in gains between periods 2 and 3. A comparison of the baseline period 1 with the last period 4 showed differences for both treatments, with a greater numerical increase in ADG in the PLY treatment.

PLY pigs were more feed efficient (feed-to-gain ratio PLY: 1.40 kg ± 0.12; CON: 1.78 kg ± 0.34; mean ± S.D.).

Average daily feed intake per pig per period did not differ between the treatments (PLY: 1.39 [1.15, 1.64], CON: 1.66 [1.41, 1.90], predicted mean [95% CIs], kg; *p* = 0.139).

PLY pigs had higher levels of triiodothyronine than CON on −1 DPI but not on other DPIs. Following the inoculation, T3 levels dropped in both treatments and remained low until 8 DPI, and gradually rose thereafter, until returned to the baseline levels on 21 DPI (Figure 7B).

### Gross lung lesions

There were no differences in the gross lung lesions between the treatments (PLY: 58.6 [48.4, 69.3], CON: 57.2 [46.6, 67.7], predicted mean [95% CIs], % of the total affected area of the lungs; *p* = 0.821).

## Discussion

This study was based on the premise that a positive emotional state is associated with a reduced susceptibility to disease during periods of increased pathogenic load [e.g., common cold in humans, (36)]. The current study explored whether rearing pigs with regular play opportunities, presumably generating positive emotions (10, 15), enhanced resilience to PRRSV infection. The results indicate that the pigs reared with play opportunities were less affected by the virus and demonstrated better performance following PRRSV infection. The play treatment pigs (PLY) were reared in standard production conditions with a chain as the only enrichment in the home pen but were provided with regular provision of periodic play opportunities from 5 days of age by access to a playpen offering extra space with various physical enrichment. In contrast, control pigs (CON) were reared conventionally without play promotion. Improved disease resilience in PLY pigs is indicated by the ability to mitigate the detrimental effects of a pathogen (37), regardless of viral load. Intriguingly, these findings underscore the value of positive experiences for farmed pigs, demonstrating their potential to enhance pigs’ resilience towards coping with various challenges encountered in modern production environments, thereby improving pig health and welfare.

There is limited, yet growing, research exploring the effects of positive affective states [e.g., positive short-term emotions and long-term moods; (38)] on health (36). Therefore, the interpretation of the results combines findings from positive and negative affective states, including different species. A simplified explanation for the beneficial effects of positive emotions on the immune system, in the case of the current study generated from play, was reviewed by Marsland et al. (39). Lymphoid organs are predominantly innervated by the sympathetic branch of the autonomous nervous system (ANS) (40), and thanks to this interplay between the ANS, the immune system, as well as positive and negative stimuli leading to emotional states, immune cells can be activated through the sympatho-adrenal-medullary (SAM) or hypothalamic–pituitary–adrenal (HPA) pathways (40). In the case of positively-valenced stimuli (e.g., play), this can lead to the detection of hormones and endogenous opioid secretion, sensitising immune cells with relevant receptors, and modifying the immune response. It has been proposed that a positive affective state could work as a stress-buffering agent, alleviating the perception of negative states and ameliorating the activation of the ANS and HPA (39). Endogenous opioids (41) involved in the reward system and released during pleasurable activities such as social play in rats (42), have anti-stress properties (43), supporting the stress-buffer theorem of the positive affective state.

In the present study, it is plausible that the pigs reared with play opportunities had modified activation of the ANS and HPA in the presence of PRRSV infection, impacting the immune response. This could be the reason leading to a lower count of immune cells, less severe respiratory distress, and an improved growth rate compared to CON pigs. Social and cognitive development can be impaired in barren housing conditions (44, 45), contributing to compromised coping when faced with stressors. Thus, CON pigs from the current study representing conventionally barren-reared pigs, might have manifested a heightened pro-inflammatory immune response to PRRSV challenge, negatively impacting disease outcomes.

Skin lesions indicate agonistic encounters (33). New pen groups formed at weaning involved the mixing of two unacquainted litters, which commonly results in more aggression to establish a new hierarchy and thus more skin lesions (46). However, the treatments did not differ in skin lesion score pre- nor one-day post-weaning. The pen groups were weaned into bigger pens [0.3 m^2^/pig in this study versus 0.18 m^2^/pig in recommended practice for 10 kg weaners; NFACC (47)], which could alleviate post-weaning aggression. Following transport, the skin lesion score increased but was substantially lower in PLY pigs compared to CON. Transport is a stressful experience for pigs, and in the current study, it also involved the mixing of two pens within-treatment. Greater exposure to play opportunities had positive effects on emotional flexibility in rats (48), and the development of more functional social dynamics in pigs (49). Gilts with more pre-weaning play-fighting experience had higher success in a contest dyad with a weight-matched (<6% weight difference) non-littermate when tested at 8 weeks of age (the opposite was seen for barrows) (49). Therefore, the play opportunities provided to PLY pigs could influence the perception of various stress-inducing environmental challenges such as transport and improve the appraisal of an agonistic situation. Rearing in larger pens with chewable enrichment (e.g., straw) supports the fulfilment of pigs’ motivation to explore (50). Thus, in the current study, PLY pigs might have spent more time exploring the transport trailer bedded with straw, and less time manipulating other pigs, as seen during transport and in lairage in Jong et al. (51). Throughout the infection, PLY pigs continued to maintain a lower skin lesion score. Disease (52) and immune stimulation (53) influenced social behaviour in pigs, resulting in more ear and tail-directed manipulative behaviours. According to Munsterhjelm et al., (52, 53), the shift in social motivation could be attributed to mounting an inflammatory response to a pathogen (54, 55), resulting in more negative affective states making the sick pigs “grumpy” (53). During the PRRSV infection in the current study, respiratory distress experienced by CON pigs was more severe and lasted longer; therefore, CON pigs could experience stronger sickness-induced irritability (54). This aligns with higher aggression and more skin lesions in CON compared to the PLY treatment, as observed until the end of the current study.

The behaviour of individual pigs sharing the same pen is not independent. However, for this study, utilising play behaviour to support resilience, it was desirable to pen pigs in larger social groups. This setup enhanced social pig behaviour and emotional contagion during play (11, 56) which naturally occurs when multiple individuals are penned together. This also better resembles an industry standard, where pigs are housed in larger groups to use space efficiently. To minimize the pen effect on behaviour, pigs could be housed with fewer pen mates, thereby increasing the number of pens while maintaining the same total number of pigs. However, this approach would reduce the study’s external validity to some extent. As this study serves as an initial investigation into the effects of play on disease resilience, it is recommended that future research increases both the sample size and the number of pens per treatment.

The treatments did not result in any changes in viral load; however, the viral load distribution in the CON treatment was more consistent, with the exception of one outlier pig, whereas the viral load in the PLY treatment exhibited greater variability. Typically, a reduction in viral load could decrease the negative economic impact on production parameters (57); however, PLY pigs achieved better performance despite having the same viral load. Housing pigs in groups can influence the spread of pathogens. However, in this study, all pigs were intramuscularly and intranasally inoculated with an equal dose of PRRSV on the same day, following a standard approach used in experimental PRRSV inoculation studies with pigs housed in groups (58). Although horizontal transmission of the virus varied among pigs, it likely had little to no impact on pathogen spread, as the pigs were already infected when shedding and horizontal transmission occurred.

The pigs in this study demonstrated a typical immune response to PRRSV, with the lowest levels of WBCs, neutrophils, lymphocytes, and monocytes in the first week post-inoculation and a rebound in the second week until euthanasia at 22 DPI. Harding et al. (59) proposed that lower immune cell numbers in blood following PRRSV infection can be due to cell trafficking from blood to the site of the infection, supporting an effective immune response to PRRSV.

The number of monocytes was lower in PLY pigs at 8 DPI, returning to baseline by 21 DPI, whereas in CON pigs, it exceeded the baseline at 21 DPI. In response to inflammation, monocytes are derived from the bone marrow into the bloodstream and secrete pro-inflammatory cytokines (60). Monocytes are refractory (61, 62) and quickly migrate into tissues, differentiating into macrophages and dendritic cells. They are particularly important for the current study since macrophages with a CD163 receptor are the primary cells supporting PRRSV replication (19) while circulating monocytes are unable to be infected (61). The increased number of monocytes in CON pigs can signify a greater inflammatory response to the virus compared to PLY. However, it can also be interpreted as a weaker immune response to PRRSV of the PLY treatment, resulting in fewer monocytes to combat the virus. Arguably, evidence shows that regular moderate exercise can have anti-inflammatory properties by upregulating a transient release of IL-6 (pro-inflammatory), which induces a subsequent increase of IL-10, IL-1 receptor antagonist (anti-inflammatory), and cortisol, as well as by reducing pro-inflammatory monocytes in the blood [for a review see (63)]. Moreover, a reduction in inflammatory monocytes (CD14+, CD16+) due to regular moderate exercise (mix of endurance and resistance training: 3×/week for 20 min for 12 weeks) was seen in healthy elderly (64). Considering that PLY pigs had more opportunities to exercise and continued to play during the infection, speculatively, the exercise could be involved in the modified immune response in the PLY treatment. Nevertheless, the results must be interpreted with caution, and a more profound analysis of the immune cells, and a quantification of cytokines to elucidate the findings is recommended.

The numbers of WBC and neutrophils were lower in PLY pigs compared to CON; however, this difference was observed only in slow-growing pigs pre-inoculation. The number of lymphocytes also tended to be lower in PLY. Importantly, the treatments did not differ in response to the infection but were different already at their baselines (−1 DPI). In the case of WBC and neutrophils, it can be assumed that CON pigs had been fighting a subclinical bacterial infection, slowing down the growth. However, the two CON pigs treated for potential bacterial infection were both from the fast-growing group pre-inoculation. Before the baseline blood collection on −1 DPI, PLY pigs had been receiving 5 weeks of daily play promotion. It has been shown that conventionally housed finisher pigs, which is equivalent to CON pigs in the current study, had a higher count of WBC, indicating greater immune activation and susceptibility to be affected by stress, compared to pigs continuously housed (16 weeks) in an environment facilitating positive experiences from interactions with straw, jute bags and wood shavings (65). This indicates that the proposed stress-buffering function of play could explain why CON pigs in the current study had a higher count of WBC, neutrophils and lymphocytes, emphasizing the reciprocal regulatory interactions between the ANS and immunity (40). Blood sampling during the pre-weaning period to establish WBC count and its differentials would provide a more specific conclusion and is recommended in future studies.

Interestingly, regarding the findings of WBC and neutrophils and their interactions with ADG pre-inoculation, the PLY treatment represented a more uniform group. This indicates that in PLY, the disease outcomes remained unaltered by individual pig-based factors. A lower variance of deviations has been proposed as a new indicator of resilience (66), suggesting that animals having more similar opportunities for recovery, regardless of their predispositions before an infection, are more resilient.

Rectal temperature (RT) did not differ in PLY and CON pigs. Similarly, RT remained similar in both pigs reared in an enriched and conventional systems after experimental PRRSV infection but was 0.16°C lower in the enriched pigs after coinfection with APP (7). On the contrary, a higher RT was seen in pigs reared in an alternative system after an LPS challenge (8). An increase in body temperature stimulates the recruitment of the immune cells and inhibits the growth of an invading microorganism, however, if the response is prolonged or too intense, it depletes the body’s resources (55). In the current study, PRRSV might elicit a uniform immune response in PLY and CON, resulting in a similar increase in rectal temperature. Additionally, the used thermometers may not be sensitive enough to detect minor differences between treatments.

PRRSV slows the growth rate by decreasing protein and lipid accretion (67, 68), negatively impacting digestibility and feed efficiency (68). PLY pigs had a higher ADG post-inoculation compared to CON. The higher ADG in PLY started from 0 DPI, with the difference being most profound in the second half of the infection. Importantly, the PLY treatment was also more feed efficient, consuming less feed per kg of gain. Mounting an immune response against infectious agents requires the remobilisation of energy and nutrients from growth and other metabolic demanding processes towards survival strategy (69). That PLY and CON pigs used energy sources for growth during the infection differently is supported by no difference in ADG pre-weaning. Additionally, the treatments did not differ in average feed intake pre- and post-inoculation; in fact, PLY pigs consumed 0.27 kg less feed on average. The improved growth in the PLY treatment aligns with their less severe clinical signs. Subtle and obvious abdominal breathing (moderate and severe respiratory distress, respectively) began to manifest in the second week post-inoculation, indicating that the pigs were clinically the sickest. However, PLY pigs were less likely to suffer from moderate and severe respiratory distress and if they did, they experienced the distress for fewer days than CON. This suggests that the PLY treatment might have had a lower inflammatory response and was able to redirect more energy towards growth instead of expending it on fighting the virus.

In the current study, PLY pigs had higher baseline levels of T3 compared to CON. Following the inoculation, the T3 pattern followed a PRRSV-typical response with an abrupt decline and rebound in the second and third weeks post-inoculation and did not differ between the treatments. Only at 21 DPI were the T3 levels numerically higher in PLY. Thyroid hormone regulates basal metabolic rate (70) and during metabolic disruptions such as disease, it controls growth and immune response (71). The depression in thyroid hormones following PRRSV infection has been proposed as a responsible agent for the decline in growth (26). Higher levels of T3 during PRRSV infection were associated with higher gains in Pasternak et al. (26); however, as seen in PLY pigs in the current study, perhaps the higher T3 levels before infection also helped to negate the suppressive effects of PRRSV on growth.

Intriguingly, PLY pigs continued to play even during the infection. One of the five play criteria defined by Burghardt (72) is that animals play in a ‘relaxed field’, meaning they are healthy and stress-free. Since play theoretically declines in adverse conditions such as injury, sickness, and hunger (12, 73, 74), it is suggested that play could serve as a fitness^4^ indicator. Locomotor play in pigs might be the most labile type when faced with a disease challenge since it involves the most activity and thus expends more energy than other play types. In the current study, following the inoculation, locomotor play decreased, and its duration remained lower compared to pre-challenge levels. On the contrary, PLY pigs played longer with objects in the second week post-inoculation than pre-inoculation, and the duration of social play fluctuated only slightly. Considering the overlap between the reward system and activated brain regions during social play in rats (13, 41), in the current study, all types of play continued to be rewarding for the pigs during the infection. However, the intensity of the most energetically demanding locomotor play was adjusted according to subjective recovery status. Considering the beneficial effects of exercise on health (63) and the increased locomotor opportunities for PLY pigs, it is currently unclear whether the observed improvement in disease resilience is due to positive emotions, physical activity, or a combination of both. Despite being sick with detected viremia and immune response, PLY pigs were motivated to play when given the opportunity. They compensated for the exerted energy by resting before and after the play sessions, as indicated by their lower activity levels compared to CON pigs on days 11 and 16 DPI. Similarly, exploration was lowest in the second week post-infection along with experiencing the highest severity of respiratory distress, indicating that the pigs did not cease rewarding behaviours but rather adjusted the intensity when faced with challenges.

Although the severity of respiratory distress differed between PLY and CON pigs, gross lung lesions did not; almost 60% of the lung area was affected in both treatments. In the study by van Dixhoorn et al. (7), differences were seen between pigs reared in an environment promoting positive experiences and those in conventional housing in a co-infection model with PRRSV and APP, where pigs in enriched pens developed fewer lung lesions and experienced less severe tissue damage than their controls. PRRSV-positive pigs are more susceptible to secondary bacterial infection due to their suppressed immune system (16) and co-infection with another bacterial pathogen [e.g., APP, *Streptococcus suis* (22), *Haemophilus (Glaesserella) parasuis* (75)] induces more severe clinical signs than PRRSV alone. Gross evaluation of PRRSV-related lung lesions might not have been sensitive enough to reveal potential subtle differences and complementary histological assessment would shed more light on the matter.

## Conclusion

Pigs reared with regular intermittent play opportunities (PLY) demonstrated modified immune, clinical, behavioural, and physiological responses in the presence or following infection with porcine reproductive and respiratory syndrome virus (PRRSV) compared to conventionally-reared pigs (CON). The evidence consistently indicates that PLY pigs were less affected by the virus and performed better than CON pigs, suggesting enhanced disease resilience. This study provides evidence that rearing pigs in an environment supportive of positive experiences by providing play opportunities can enhance resilience against common challenges in modern pig production, supporting the value of positive welfare for intensively farmed pigs.

## Data Availability

The original contributions presented in the study are publicly available. This data can be found here: https://doi.org/10.5061/dryad.76hdr7t55.

## Footnotes

^1^Frequency of play within the first 5-min of the play session at the age of 21 days: 98% of PLY pigs played at least once and were considered for the selection. However, because 83% of PLY pigs played at least 5x, pigs showing play behaviour more frequently, were preferred.

^2^Aggression typically does not result in tail lesions, meaning the tail generally does not show lesions with scores of 1–3. Therefore, the tail should not have been included in the skin lesion scoring. However, it was included because tail lesions were infrequent and preliminary analysis found no significant differences between treatments on any given day (*p* > 0.05, Fisher’s exact test).

^3^As determined during necropsy, the cause of death in the deceased pig was identified through visual examination of lung lesions, revealing a potential presence of either *Streptococcus suis* or *Haemophilus parasuis bacteria*. However, as no tissue was collected during necropsy, bacterial culture was not conducted.

^4^Crude definition: *“Fitness involves the ability of organisms—or, more rarely, populations or species—to survive and reproduce in the environment in which they find themselves.”* (76).
