# Supplementary material for: Rearing pigs with play opportunities: the effects on disease resilience in pigs experimentally inoculated with PRRSV
Source: Front Vet Sci. 2024 Sep 17;11:1460993. doi: 10.3389/fvets.2024.1460993 (PMC11443507; doi:10.3389/fvets.2024.1460993)
Supplement: Supplementary file 1 [file Table_1.docx]

## Supplementary material

**Supplementary Table 1.** Clinical signs of porcine reproductive and respiratory syndrome virus with scores based on severity recorded in the AM and PM on experimentally inoculated pigs at biosecurity level 2 and negative control pigs at biosecurity level 1 during the acclimation and post-inoculation period.

| Clinical signs | Score | Description |
| --- | --- | --- |
| Respiratory distress (RD) | | |
|  | 0 | None: Normal respiratory rate (25-40 breaths per min, bpm) |
|  | 1 | Mild: No abdominal effort but increased respiratory rate (>40 bpm) |
|  | 1.5 | Moderate: Slight abdominal effort (very careful observation required of the pig's abdomen slightly moving up and down as this is not noticeable at first sight) and normal respiratory rate (25-40 bpm) |
|  | 2 | Moderate: Slight abdominal effort (very careful observation required of the pig's abdomen slightly moving up and down as this is not noticeable at first sight) and increased respiratory rate (>40 bpm) |
|  | 2.5 | Severe: Obvious abdominal breathing (dyspnoea; fairly obvious to an untrained eye but not heavy thumping) but the normal respiratory rate (25-40 bpm) |
|  | 3 | Severe: Obvious abdominal breathing (dyspnoea; fairly obvious to an untrained eye but not heavy thumping) and increased respiratory rate (>40 bpm) |
| Coughing | | |
|  | 0 | None |
|  | 1 | Sporadic coughs, easily countable, generally less than 10 in 3 min |
|  | 2 | Short bouts of coughing lasting about 5-10 sec, not more frequent than 1 bout/min, followed by long periods with no coughing |
|  | 3 | Repeated short bouts (>1 per min) at irregular intervals separated by short periods without coughing |
|  | 4 | Continual or repeated coughing (not bouts) for more than 15 sec |
| Responsiveness | | |
|  | 0 | Alert and active, pays attention to the stock person |
|  | 1 | Alert, but slower than pen mates |
|  | 2 | Reluctant to move, but gets up by stimulation |
|  | 3 | Down, does not respond to stimulation or demonstrates seizures (leg paddling, recumbency) |
| Appetite | | |
|  | 0 | Normal appetite - feed use is apparent, pig appears satisfied, maintaining weight |
|  | 1 | Reduced feed intake - pig may show hollowing on flanks and slender stomach suggestive of lower gut fill |
|  | 2 | Anorexic |
| Colour of the skin | | |
|  | 0 | Normal |
|  | 1 | Small area (<50%) of subcutaneous hyperaemia on the ear, tail, or abdomen |
|  | 2 | >50% of the ear, tail or abdomen have hyperaemia or cyanosis, no obvious necrosis |
|  | 3 | >50% of the ear, tail or abdomen have hyperaemia or cyanosis with necrosis of the skin |
| Consistency of the faeces | | |
|  | 0 | Formed, normal |
|  | 1 | Wet cement or loose cow pie |
|  | 2 | Runny |
|  | 3 | Mucoid |
|  | 4 | Bloody or reddish coloured with or without mucoid |
| Body condition | | |
|  | 0 | Normal body condition (score 3; NFACC, 2014) and gut fill |
|  | 1 | Normal body condition (score 3; NFACC, 2014), hollow flanks, hairy |
|  | 2 | Slight loss of body condition, backbone evident, weight loss up to 15% of body weight |
|  | 3 | Moderate loss of body condition, backbone prominent, weight loss greater than 15% of body weight |

- Lameness was monitored as a score of 0 (none), 1 (sore/swollen, but weight-bearing), 2 (sore/swollen, and non-weight-bearing), and 3 (unable to walk, recumbent).


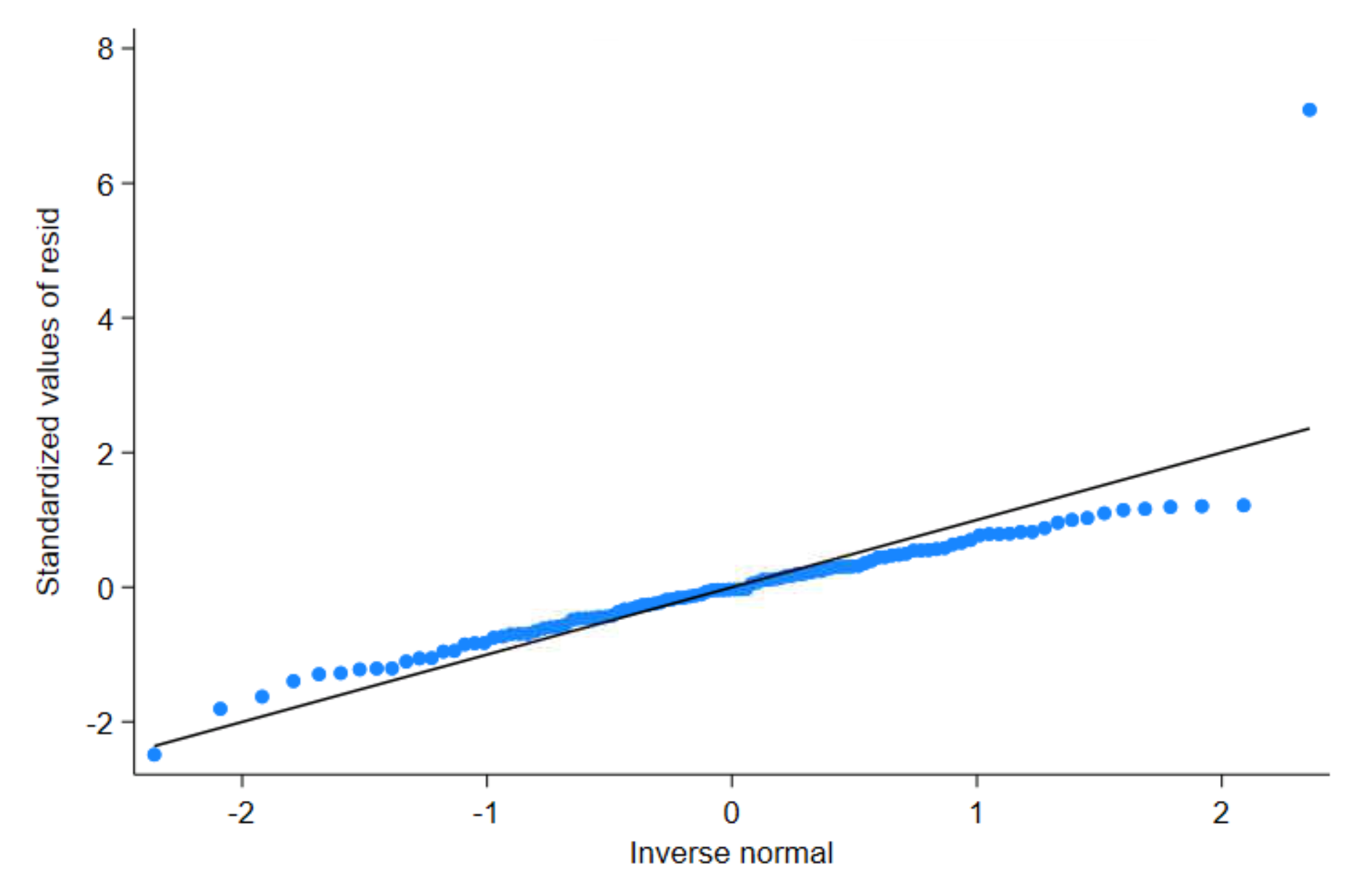


**Supplementary Figure 1**. Scatter plot of the standardized residuals for final model of ADG post-inoculation (fixed effects of treatment, period, and interaction effect of treatment-period, pig in the random statement) depicting the excluded outlier from the Control treatment from DPI period 4 in a red square.

**Supplementary Table 2**. Reference intervals for porcine cell blood count from Prairie Diagnostic Services Inc. (address: 52 Campus Dr, S7N 5B4, Saskatoon, SK, Canada).

| Cell type | Reference interval ×10^9^/L |
| --- | --- |
| White blood cells | 11.0 - 22.0 |
| Neutrophils | 3.080 - 10.400 |
| Lymphocytes | 4.290 - 13.600 |
| Monocytes | 0.220 - 2.200 |

**Supplementary Table 3**. Proportion of Play (PLY) and Control (CON) treatment pigs in the numbers of white blood cells (WBC), lymphocytes, monocytes, and neutrophils outside/within the reference interval (suppl. Table 2) at -1, 2, 4, 8, 13, 17, and 21 days post-inoculation (DPI). Results are expressed as % of pigs per treatment (number of pigs) outside/within of the reference interval.

| Cell blood count | DPI | Within reference interval  % (n) | | Outside reference interval  % (n) | |  |
| --- | --- | --- | --- | --- | --- | --- |
|  |  | CON | PLY | CON | PLY | p-value |
| WBC | -1 | 78.6 (11) | 85.7 (12) | 21.4 (3) | 14.3 (2) | 1 |
|  | 2 | 78.6 (11) | 69.2 (9) | 21.4 (3) | 30.7 (40 | 0.68 |
|  | 4 | 71.4 (10) | 50.0 (7) | 28.6 (4) | 50.0 (7) | 0.44 |
|  | 8 | 85.7 (12) | 84.6 (11) | 14.3 (2) | 15.4 (2) | 1 |
|  | 13 | 57.1 (8) | 53.9 (7) | 42.9 (6) | 46.15 (6) | 1 |
|  | 17 | 42.9 (6) | 76.9 (10) | 57.1 (8) | 23.1 (3) | 0.12 |
|  | 21 | 42.9 (6) | 84.6 (11) | 57.1 (8) | 15.4 (2) | 0.05 |
| Lymphocytes | -1 | 85.7 (12) | 92.9 (13) | 14.3 (2) | 7.1 (1) | 1 |
|  | 2 | 100. 0 (14) | 100.0 (12) | 0 | 0 | - |
|  | 4 | 92.9 (13) | 92.9 (13) | 7.1 (1) | 7.1 (1) | 1 |
|  | 8 | 100. 0 (14) | 100.0 (13) | 0 | 0 | - |
|  | 13 | 78.6 (11) | 92.3 (12) | 21.4 (3) | 7.7 (1) | 0.6 |
|  | 17 | 64.3 (9) | 92.3 (12) | 35.7 (5) | 7.7 (1) | 0.17 |
|  | 21 | 57.1 (8) | 76.9 (10) | 42.9 (6) | 23.1 (3) | 0.42 |
| Monocytes | -1 | 100. 0 (14) | 100.0 (14) | 0 | 0 | - |
|  | 2 | 50.0 (7) | 66.7 (8) | 50.0 (7) | 33.3 (4) | 0.45 |
|  | 4 | 78.6 (11) | 71.4 (10) | 21.4 (3) | 28.6 (4) | 1 |
|  | 8 | 100.0 (14) | 84.6 (11) | 0 | 15.4 (2) | 0.22 |
|  | 13 | 100.0 (14) | 100.0 (13) | 0 | 0 | - |
|  | 17 | 100.0 (14) | 100.0 (13) | 0 | 0 | - |
|  | 21 | 100.0 (14) | 100.0 (13) | 0 | 0 | - |
| Neutrophils | -1 | 85.7 (12) | 92.9 (13) | 14.3 (2) | 7.1 (1) | 1 |
|  | 2 | 71.4 (10) | 58.3 (7) | 28.6 (4) | 41.7 (5) | 0.68 |
|  | 4 | 78.6 (11) | 71.4 (10) | 21.4 (3) | 28.6 (4) | 1 |
|  | 8 | 85.7 (12) | 92.3 (12) | 14.3 (2) | 7.7 (1) | 1 |
|  | 13 | 78.6 (11) | 84.6 (11) | 21.4 (3) | 15.4 (2) | 1 |
|  | 17 | 78.6 (11) | 76.92 (10) | 21.4 (3) | 23.1 (3) | 1 |
|  | 21 | 92.9 (13) | 100.0 (13) | 7.1 (1) | 0 | 1 |


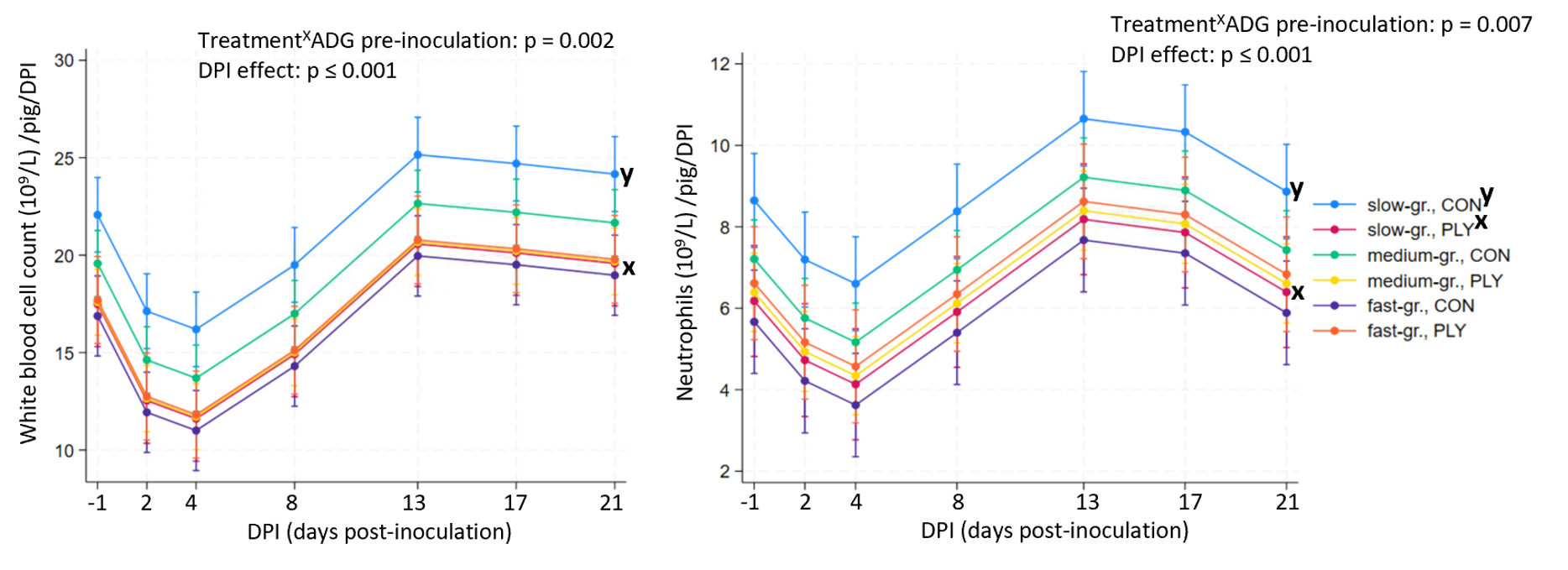


**Supplementary Figure 2.** Total count of white blood cells (WBC) and neutrophils in slow-, medium- and fast-growing (gr.) pigs pre-inoculation (WBC and neutrophils, 10^9^/L; a, b, respectively) in Play (PLY) and Control (CON) treatments per pig (n = 28) on -1, 2, 4, 8, 13, 17 and 21 days post-inoculation (DPI). Data are presented as predicted means and 95% confidence intervals.

[Footnote: ‘X’ between two variables signifies an interaction effect. Significant differences between treatments within weight category (a, b) are denoted on the graph legends and next to the relevant lines with a superscript letter in bold (x, y). Weight categories based on ADG pre-inoculation: slow- (n = 4 PLY, 5 CON pigs, mean = 0.28, range = 0.26 – 0.28; kg), medium- (n = 7 PLY, 3 CON pigs, 0.30, 0.29 – 0.31) and fast-growing (n = 3 PLY, 6 CON pigs, 0.33, 0.32 – 0.34) pigs pre-inoculation. Graphs a, b: No interaction effect of treatment with time variable is present, thus, the presented values are summarized over all values (both treatments show the same DPI to DPI change). The pairwise comparisons listed below (in bold) have p-values less than or equal to the significant threshold (ST), adjusted using the Bonferroni correction to control the analysis-wise error. The type and number of comparisons are in italics and parentheses, respectively. **a: DPI effect** - *across consecutive days, baseline DPI-1 vs 21*. ST (7): p = 0.007. DPI-1 vs 2, DPI4 vs 8, DPI8 vs 13, DPI-1 vs 21. **b: DPI effect** - *across consecutive days, baseline DPI-1 vs 21.* ST (7): p = 0.007. DPI-1 vs 2, DPI4 vs 8, DPI8 vs 13, DPI17 vs 21.]

**Supplementary Table 4.** The negative control pigs and their rectal temperature (RT, °C), weight (kg), triiodothyronine (T3, nmol/L), the number of white blood cells (WBC, 10^9^/L), neutrophils (neutr., 10^9^/L), lymphocytes (lymph., 10^9^/L), monocytes (mono., 10^9^/L) and their relative percentage (%) at 0 (RT, weight) or -1 (blood cells), 13 and 21 days post-inoculation (DPI).

[Footnote: *ADG between 0 and 21 DPI pig 15: 1.0 kg; pig 30: 0.7 kg. ^+^ sample lost]

| Pig ID | DPI | RT (°C) | Weight (kg)* | T3 (nmol/L) | WBC (10^9^/L) | Neutr. (10^9^/L) | % Neutr. | Lymph. (10^9^/L) | % Lymph. | Mono. (10^9^/L) | % Mono. |
| --- | --- | --- | --- | --- | --- | --- | --- | --- | --- | --- | --- |
| 15 | 0/-1 | 40.1 | 13.5 | 1.66 | 17.6 | 5.6 | 32 | 10.2 | 58 | 1.1 | 6 |
| 30 | 0/-1 | 39.6 | 11.5 | 0.98 | 21.1 | 9.1 | 43 | 11.2 | 53 | 0.6 | 3 |
| 15 | 13 | 39.8 | 25.3 | 1.33 | 14.9 | 4.2 | 28 | 10.1 | 68 | 0.4 | 3 |
| 30 | 13 | 39.4 | 19.2 | 1.28 | missing^+^ | missing | missing | missing | missing | missing | missing |
| 15 | 21 | 39.2 | 33.5 | 1.54 | 16.1 | 1.8 | 11 | 13.7 | 85 | 0.3 | 2 |
| 30 | 21 | 39.6 | 25.9 | 1.44 | 16.1 | 6.0 | 37 | 9.2 | 57 | 0.6 | 4 |

**Supplementary Section 1.** Significant main effects (p ≤ 0.05) not relevant to treatment differences.

Skin lesion score:

- Sex effect (p = 0.004): Barrows had a lower skin lesion score than gilts (Barrows; B: 6.94 [6.47,7.42], Gilts; G: 7.95 [7.45, 8.45], predicted mean [95% CIs], max score 18/pig; p = 0.004).

Monocytes:

- ADG pre-inoculation (p = 0.007).

Rectal temperature (RT):

- ADG pre-weaning (p = 0.019).

Feeding bouts AM&PM:

- ADG pre-weaning (p = 0.015).
- DPI effect (p = 0.056): Pigs had fewer feeding bouts in the first (7 DPI) and second week (11, 16 DPI) post-inoculation compared to pre-inoculation, but bounced back to baseline levels at the end of the trial (20 DPI) (-2 DPI: 2.71 [2.10, 3.31], 3 DPI: 2.06 [1.53, 2.56], 7 DPI: 1.71 [1.23, 2.20], 11 DPI: 1.80 [1.29, 2.30], 16 DPI: 1.89 [1.35, 2.39], 20 DPI: 2.48 [1.89, 3.07], predicted count [95% CIs], frequency/DPI; p = 0.056)

Triiodothyronine (T3):

- Sex effect (p = 0.001): Barrows had a lower T3 levels than gilts (B: 0.86 [0.78, 0.93], G: 1.04 [0.97, 1.12], predicted mean [95% CIs], nmol/L; p = 0.001).

Average daily feed intake per pig per DPI period:

- DPI effect (p ≤ 0.001). Average daily feed intake per pig increased over time and was highest in the end of the experiment (pre-inoc.: 0.69 [0.44, 0.95], 0 to 6 DPI: 1.04 [0.79, 1.29], 7 to 13 DPI: 1.07 [0.82, 1.32], 14 to 21 DPI: 3.26 [3.04, 3.55], predicted mean [95% CIs], kg; p ≤ 0.001).

**Supplementary Section 2.** List of clinical signs experienced by Play (PLY) and Control (CON) treatment pigs post-inoculation with obtained scores and ranges of days.

- Responsiveness: Eight PLY and nine CON pigs showed decreased responsiveness (score ≥ 1) for at least one day post-inoculation. Six PLY pigs showed decreased responsiveness (score ≥ 1) compared to one CON pig at 1 DPI and zero CON pigs and 2 DPI (1 DPI: p = 0.038, 2 DPI: p = 0.008; Fisher’s exact). Eight PLY and CON pigs were ‘alert, but slower than pen mates’ (score 1; range: 1 – 8 days). Five PLY pigs were ‘reluctant to move but got up by stimulation’ for more than one day (score 2; range: 1 – 5 days) and one PLY pig ‘did not respond by stimulation’ (score 3) for one day, while four CON pigs had score 2 for one day.
- Coughing: Nine PLY pigs (range: 1 – 4 days) and eight CON pigs (range: 1 – 3 days) had sporadic coughs, easily countable, generally less than 10 in 3 min (score 1) or short bouts of coughing lasting about 5-10 sec, not more frequent than 1 bout/min, followed by long periods with no coughing (score 2).
- Skin colour: Seven PLY (range: 1 – 3 days) and nine CON pigs (range: 1 – 7 days) experienced subcutaneous hyperaemia on small area (<50%) on the ear, tail, or abdomen. One CON pig had hyperaemia or cyanosis on >50% of the ear, tail or abdomen have with no obvious necrosis (score 2) for one day.
- Body condition: Ten PLY pigs had ‘normal body condition, hollow flanks, hairy’ (score 1) compared to five CON pigs at 13 DPI (p = 0.031, Fishers’ exact). Twelve PLY (range: 3 – 19 days) and nine CON pigs (range: 2 – 22 days) had ‘normal body condition, hollow flanks, hairy’ (score 1).
- Appetite: Three PLY pigs (range: 1 – 2 days) and two CON pigs (1 day) had depressed appetite showing hollowing on flanks and slender stomach suggestive of lower gut fill (score 1).
- Lameness: One CON pig had one foot ‘sore or swollen but weight-bearing to some extent’ (score 1) from 2 to 9 DPI.

**Supplementary Section 3.** Additional details about model outputs indicating differences between Play (PLY) and Control (CON) treatment pigs inoculated with PRRSV (sections a, b, c; n = 28) and only in the PLY pigs (section d; n = 14):

a) PRRSV RNA concentration under the curve (AUC) and its predicted means [95% confidence intervals] and p-value:

- All pigs included (n=28): PLY: 1.84e+12 [1.17e+12, 2.51e+12], CON: 1.41e+12 [7.38e+11, 2.08e+12], RNA copies/mL; p = 0.362
- The outlier CON pig excluded (n=27): PLY: 1.84e+12 [1.29e+12, 2.39e+12], CON: 1.13e+12 [5.63e+11, 1.70e+12], RNA copies/mL; p = 0.077

b) Medical treatments treating rectal temperature (RT) exceeding 40°C:

A medical treatment to lower the RT exceeding 40°C (Metacam®, 0.4 mg/kg) was administered to three PLY (1x gilt LR fast-gr. pre-inoc., 1x barrow MR fast-gr. pre-inoc., 1x barrow MR slow-gr. pre-inoc.) and two CON pigs (1x gilt HR, 1x barrow HR, both fast-gr. pre-inoc.), respectively, between 7 to 13 DPI. The same two CON pigs also received antibiotics to treat potential secondary infection (EXCENEL® RTU EZ, ceftiofur hydrochloride; 3 to 5 mg CE/kg; ZOETIS INC., QC, Canada) between 9 to 12 DPI. As analyzed in mixed multilevel univariable linear regression, the RT was not affected by the administration of Metacam (p = 0.301) or the antibiotics (p = 0.572).

c) Active behaviour in the AM and PM:

In the AM, PLY pigs were less active than CON on 11 and 16 DPI. In PLY, active behaviour in the AM did not differ over time, but it increased in CON between 11 and 16 DPI. Neither of the treatments differed in the AM active behaviour from baseline on 21 DPI (suppl. mat. Figure C). In the PM, PLY was less active than CON (PLY: 2.87 [0.67, 1.07], CON: 2.92 [2.55, 3.30], predicted count [95% CIs], scans/pig; p ≤ 0.001). The PM active behaviour decreased over time (-2 vs 21 DPI p ≤ 0.001, ST (6): p ≤ 0.008).


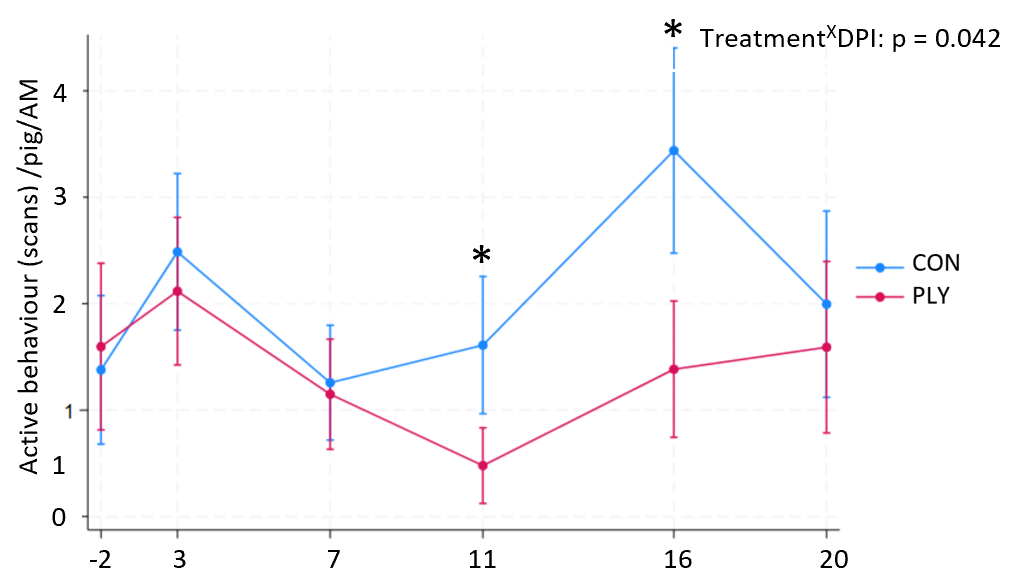


Supplementary Figure C. in section 3. Active behaviour in the AM in Play (PLY) and Control (CON) treatments per pig (n = 28) within the initial 10 min of a play session on -2, 3, 7, 11, 16 and 20 days post-inoculation (DPI). Data are presented as predicted counts and 95% confidence intervals.

[Footnote: ‘X’ between two variables signifies an interaction effect. Significant differences between the treatments within DPI are denoted on the graph with an asterisk (*).]

d) Model fit (Akaike information criterion; AIC) from the final multilevel regression models with negative binominal distribution of object, locomotor and social play and exploratory behaviour:

- object play: 835.241
- locomotor play: 750.924
- social play: 578.331
- exploratory behaviour: 923.619
